# High GUD Incidence in the Early 20^th^ Century Created a Particularly Permissive Time Window for the Origin and Initial Spread of Epidemic HIV Strains

**DOI:** 10.1371/journal.pone.0009936

**Published:** 2010-04-01

**Authors:** João Dinis de Sousa, Viktor Müller, Philippe Lemey, Anne-Mieke Vandamme

**Affiliations:** 1 Laboratory for Clinical and Evolutionary Virology, Rega Institute for Medical Research, Katholieke Universiteit Leuven, Leuven, Belgium; 2 Institute of Biology, Eötvös Loránd University, Budapest, Hungary; 3 Centro de Malária e Outras Doenças Tropicais, Instituto de Higiene e Medicina Tropical, Universidade Nova de Lisboa, Lisboa, Portugal; Institute of Infectious Disease and Molecular Medicine, South Africa

## Abstract

The processes that permitted a few SIV strains to emerge epidemically as HIV groups remain elusive. Paradigmatic theories propose factors that may have facilitated adaptation to the human host (e.g., unsafe injections), none of which provide a coherent explanation for the timing, geographical origin, and scarcity of epidemic HIV strains. Our updated molecular clock analyses established relatively narrow time intervals (roughly 1880–1940) for major SIV transfers to humans. Factors that could favor HIV emergence in this time frame may have been genital ulcer disease (GUD), resulting in high HIV-1 transmissibility (4–43%), largely exceeding parenteral transmissibility; lack of male circumcision increasing male HIV infection risk; and gender-skewed city growth increasing sexual promiscuity. We surveyed colonial medical literature reporting incidences of GUD for the relevant regions, concentrating on cities, suffering less reporting biases than rural areas. Coinciding in time with the origin of the major HIV groups, colonial cities showed intense GUD outbreaks with incidences 1.5–2.5 orders of magnitude higher than in mid 20^th^ century. We surveyed ethnographic literature, and concluded that male circumcision frequencies were lower in early 20^th^ century than nowadays, with low rates correlating spatially with the emergence of HIV groups. We developed computer simulations to model the early spread of HIV-1 group M in Kinshasa before, during and after the estimated origin of the virus, using parameters derived from the colonial literature. These confirmed that the early 20^th^ century was particularly permissive for the emergence of HIV by heterosexual transmission. The strongest potential facilitating factor was high GUD levels. Remarkably, the direct effects of city population size and circumcision frequency seemed relatively small. Our results suggest that intense GUD in promiscuous urban communities was the main factor driving HIV emergence. Low circumcision rates may have played a role, probably by their indirect effects on GUD.

## Introduction

Independent simian immunodeficiency virus (SIV) transfers to humans have established twelve different known human immunodeficiency virus (HIV) groups [1]–[10]. Pandemic HIV-1 group M, and group N descend from SIVcpz endemic in West Central African chimpanzees [1]–[5], while the closest relatives of HIV-1 groups O and P are SIVs infecting western lowland gorillas (SIVgor) in the same region [1], [10], [11]. All known HIV-2 groups (A–H) descend from SIVsmm endemic in sooty mangabeys [4]–[9], which inhabit a strip of forested coast in West Africa [9], [12], [13].

Only four of these twelve strains generated successful epidemics in humans: HIV-1 groups M and O, and HIV-2 groups A and B. The pandemic group M strain clusters most closely with SIVcpz endemic in wild chimpanzees from the southeast corner of Cameroon [2]. There is compelling evidence, both from serology and AIDS cases, that HIV-1 infections were initially restricted to the Democratic Republic of Congo (DRC) [13], [14]. The geographical origin of the simian ancestor of HIV-1 group O is still unknown, but its human epicenter was Cameroon, a country to which it remains largely restricted [15]. Both HIV-2 groups A and B cluster more closely with SIVsmm from sooty mangabeys living in the forests of southwestern Côte d'Ivoire [9]. Both groups spread abundantly in this country, and have spread only recently to Guinea, Sierra Leone, and Liberia, while group A was able to spread to Guinea-Bissau early in its epidemic history [13], [16]. This suggests that Côte d'Ivoire was the main early epicenter of these HIV-2 groups. Of the four main groups, HIV-1 group O is the most confined; it currently infects only tens of thousands of people, mainly in Cameroon and Nigeria [15], [17], [18]. HIV-1 group N is much rarer and restricted to Cameroon [19] and the remaining HIV groups have been found in just one patient [3], [4], [7]–[10], including the recently identified HIV-1 group P strain [10]. See Figure S1 for an illustration of HIV biogeography.

Transmission of simian retroviruses to humans is not exceptional. Simian foamy viruses (SFV) have frequently been transmitted to humans exposed to bushmeat, apparently without further spread [20] and the epidemic human T-cell lymphotropic viruses (HTLV) arose from their simian counterparts (STLV) through contacts over thousands of years. It is generally accepted that SFV, STLV, as well as SIV, entered the human population through bushmeat handling. Although such events are common today [20]–[22], and therefore assumed to also have been common in the recent and distant past, they seldom result in a virus with epidemic potential. Despite progress in identifying SIVs closely related to HIV groups [1]–[3], [9], *how* and *why* only some of the transmitted SIV strains established epidemics is subject of ardent debate [3]–[5], [23], [24].

The estimated times of cross-species transmission to humans of the different HTLV-1 subtypes span between less than 3,000 and up to about 50,000 years ago [25], [26], while HTLV-2 was transferred to humans between 60,000 and 400,000 years ago [22], [25], [26]. In contrast, all main epidemic HIV groups started to spread in humans only recently, and nearly simultaneously, in the early 20^th^ century [6], [27]–[31]. This fact prompted the search for factors driving HIV emergence, which can be attributed to phenomena new in the 20^th^ century. As one of many speculative theories, a hypothesis involving SIV-contaminated polio vaccines has been extensively debated [13], but decisively refuted by many lines of scientific evidence [2]–[3], [6], [27]–[31]. Another hypothesis proposes that unsterile injections serially transmitted SIV from a bushmeat handler to other humans in a chain of acutely infected people, improving its adaptation to the new host [4], [23], [24]. Also hunting intensification [5], social changes, urbanization, and/or increased human mobility [3] have been invoked as explanations for HIV emergence.

It is conceivable that host or circumstantial factors currently increasing the transmissibility of HIV were also involved in its origin and initial spread. This notion is implicit in the theories that proposed unsterile injections as the driving factor [4], [23], [24]. Standard per-reuse transmissibility of unsterile intravenous injections is considerably higher than standard per-act sexual transmissibility (0.6–1.6% vs. 0.05–0.1%) [32], [33]. However, the involvement of genital ulcer disease (GUD) dramatically raises the latter. GUD-assisted per-act transmissibilities for HIV-1 were estimated at: 1) man to woman with GUD: 7.4% (95% CI 3.8–15.7%) [33]; 2) woman with GUD to man: 16% (95% CI 6–32%) in a cohort in which two thirds of the men were circumcised; the measured per-act transmissibility was 4% for the circumcised men, and 43% for the uncircumcised [33], [34]. Although the per-exposure risk for men with GUD has not been measured, evidence from observational studies also suggest a very high HIV transmission risk [35], [36]. The effect of GUD infections is also very strong at the population level: more than half of new HIV infections in Africa might be attributed to GUD facilitated transmission [37]. However, to our knowledge no study has investigated the role of GUD prevalence in the origin, initial spread and adaptation of HIV. Another host factor linked with HIV transmissibility is circumcision. For heterosexual men, per-act risk of HIV-1 acquisition is about 6–11 times higher if they are uncircumcised [34], [38], although more moderate odds ratios have been estimated over longer periods. For uncircumcised men exposed to GUD-suffering sex workers, the measured per-act risk was 43% [34]. Second to transfusions, this is the highest HIV transmissibility ever measured.

Here, we aimed to identify which factors could have favored SIV adaptation to humans and facilitated its emergence and spread as HIV. First, we investigated the timing of the splits between HIV-1 groups M and O, and HIV-2 groups A and B and their respective closest SIV lineage, either by reviewing the literature or by using phylogenetic methods to narrow down the missing links. Having established a likely time interval of cross species transmission, we then reviewed colonial medical, and demographic literature, including original archival sources, to investigate how the proposed risk factors, including GUD incidence, city growth, health systems, gender distribution, and commercial sex work (CSW), varied in time and space, across the relevant African regions. Additionally we reviewed ethnographic literature on male circumcision per ethnic group, and assessed whether its geographical distribution, in early 20^th^ century, overlapped with the putative epicenters for the HIV groups. Finally, we developed computer simulations based on detailed population, sociological and medical data found in our literature and archival searches to identify the key factors that might have facilitated the emergence of HIV-1 group M. Since spatial and temporal coincidence have previously been considered as evidence for factors involved in the emergence of a pathogen [39], including drafting hypotheses on the origins of HIV [3]–[5], [13], [23], [24], we here invoke such coincidences to support our hypothesis on the origin of the HIV groups.

## Results

### Estimating divergence times between HIV-1 groups M/O and their simian ancestors

We obtained divergence dates between epidemic HIVs and their closest simian relatives including recently discovered simian strains [1]–[2], through literature survey [6], [27]–[31] or by estimating new dates. We infer divergence dates using Bayesian relaxed clock analysis [40] for two separate data sets representing HIV-1 group M/SIVcpz and HIV-1 groupO/SIVgor/SIVcpz respectively.

The combined results are listed in Table 1. In general, epidemic HIV crossed to humans after the 18^th^ century (Table 1). The interspecies transmission of each HIV group occurred between the split with the closest SIV lineage and the time to the most recent common ancestor (MRCA; TMRCA) of the group, probably closer to the latter.

**Table 1:** TMRCA estimates for HIV groups and their divergence from the most closely related SIV strains.

Calculation	Dating estimates	References
**HIV-1 group M**		
Group TMRCA	1920 (1902–39)^a^ 1937 (1925–49)^a^	Salemi et al. (2000) [27]
Group TMRCA	1931 (1915–41)	Korber et al. (2000) [28]
Group TMRCA	1921 (1908–33)^b^ 1902 (1873–1922)^b^ 1908 (1884–1924)^b^	Worobey et al. (2008) [29]
Split from closest SIV	1853 (1799–1904)	Wertheim & Worobey (2009) [31]
Split from closest SIV	1876 (1847–1907)	This study
**HIV-1 group O**		
Group TMRCA	1920 (1890–1940)	Lemey et al. (2004) [30]
Group TMRCA	1905 (1866–1938)	Wertheim & Worobey (2009) [31]
Split from closest SIV	1741 (1606–1870)	This study
**HIV-1 group N**		
Group TMRCA	1963 (1948–77)	Wertheim & Worobey (2009) [31]
**HIV-2 group A**		
Group TMRCA	1940 (1924–56)	Lemey et al. (2003) [6]
Group TMRCA	1932 (1906–55)	Wertheim & Worobey (2009) [31]
Split from closest SIV	1889 (1856–1922)	Lemey et al. (2003) [6]
**HIV-2 group A**		
Group TMRCA	1945 (1931–59)	Lemey et al. (2003) [6]
Group TMRCA	1935 (1907–61)	Wertheim & Worobey (2009) [31]
Split from closest SIV	1889 (1856–1922)	Lemey et al. (2003) [6]

Mean estimates and 95% credible/confidence intervals for group TMRCAs were obtained from previous studies, whereas divergence times from the closest SIVs were estimated in this study, and taken from [31].

^**a**^the two estimates correspond to different genes used.

^**b**^The three estimates correspond to different coalescent tree priors used.

### The early 20^th^ century constituted a preferential time window for HIV emergence

The five HIV groups represented in Table 1 are the ones for which an ongoing epidemic is demonstrated; all the others have only been found in a single person [3], [4], [7]–[10]. Of these five, four (HIV-1 groups M and O, and HIV-2 groups A and B) have been able to spread at an epidemic level and are currently infecting at least tens of thousands of people, and likely adapted to humans and started to spread in early 20^th^ century, whereas HIV-1 group N may have started to spread in mid century (Table 1). Thus, the early 20^th^ century seems to have constituted a particularly permissive time window for SIV zoonoses with clear epidemic consequences; for example, both epidemiological evidence and population size studies indicate that HIV-1 group M as well as HIV-2 group A spread epidemically at rates nearly equivalent to a tenfold increase in each decade [6], [13], [41].

The narrow time interval in which the four major HIV groups emerged, contrasting with the origins of HTLVs [22], [25], [26], suggests that driving factors specific to early 20^th^ century have assisted HIV emergence in our species. The prevailing theories would predict more HIV groups emerging after 1950 than before. Injection intensity was much higher in mid 20^th^ century than before [4]. Urbanization and traffic have also intensified since mid century [5], [42]; for example, among the rural Ngbaka-Mabo people of Lobaye, in southwest Central African Republic (CAR), hunting practice was common, and by 1957, the majority of men, many of them hunters/bushmeat handlers, had already migrated to one or more large Central African cities (e.g., Bangui, Brazzaville) [42]; other rural peoples also migrated to cities abundantly, albeit not necessarily to the same extent as the Ngbaka-Mabo. The mid century also likely generated increased human exposures to SIV, and particularly SIVcpz, because the main wild chimpanzee population collapse, partly due to intensified hunting, happened between 1946 and 1980 [43], [44].

To understand why only the early 20^th^ century generated all epidemic HIVs, we aimed to reveal the full spectrum of factors that might have had the potential to increase SIV/HIV transmissibility and adaptation in the established critical time interval. In order to be consistent with a causal relationship, the factor or factors responsible should coincide both spatially and temporally with the origin of the epidemic [39], and thus should have peaked in early 20^th^ century in the geographic areas coinciding more or less with the ranges of the relevant SIV-carrying primates.

### The origin of epidemic HIVs coincided with the peak of GUD epidemics

We reviewed colonial medicine articles, reports, and reviews, for the countries of chimpanzee and sooty mangabey ranges, searching for information about sexually transmitted diseases (STDs), GUDs, and diseases requiring intensive injection treatments (see Materials and Methods, section GUD incidences survey). We found that the most commonly reported GUDs were syphilis, chancroid (*chancrelle*, *chancre mou*), and to a lesser extent, lymphogranuloma venereum (LGV) (*bubon vénérien*).

Primary and secondary syphilis (PSS) last a total of about five months, with exudative genital ulcers being present 30% of the time in either stage. This is followed by latent and tertiary stages, with no genital ulceration, and no infectiousness [45], [46]. As an epidemic progresses, a decreasing fraction of all syphilis infections are PSS; the latent and tertiary stages predominate [45], [47], [48]. Chancroid's single chancre lasts ten weeks on average [49]. Syphilis' and chancroid's high per-contact infectiousness [45], [46], [49] promotes rapid spread and high frequency of genital ulcers in local sexually promiscuous settings (e.g., PSS may attain frequencies of 20–60% during initial invasion [45]). These conditions, particularly if occurring in populations with many uncircumcised men, constitute a favorable setting for SIV adaptation to humans through serial sexual transmission during acute infection.

In the relevant regions, the early 20^th^ century witnessed very high GUD incidences especially in fast growing cities and socially changed semi-rural areas. This trend started around 1885, when European powers decidedly rushed to control the interior. Many sources explicitly state that syphilis was absent from nearly all forested areas where chimpanzees, gorillas, and sooty mangabeys live, up to 1885 [50]–[54], although it was present before in seaports with European presence [52], [55], [56], and in savannah-forest interface regions connected with Arab states [50]. Yaws (*Treponema pallidum pertenue*) has a longstanding and high prevalence in these forests [47], [52], and exhibits cross-immunity with syphilis (*Treponema pallidum pallidum*) [52]. However, this is not the explanation of why syphilis did not generate epidemics there during centuries. Indeed, these populations *did* experience epidemic syphilis, when they were recruited to cities, and when social disruption due to colonial practices entered deep in the yaws-riddled forests (e.g., in the networks of posts in the Ogooué (Gabon) and Sangha (French Congo) riversides, in the Équateur province (Belgian Congo), and in southern Cameroon [47], [54], [57]–[59]). Recent simulations show that syphilis epidemics are very dependent on highly promiscuous minorities [46]. Chancroid is also very dependent on CSWs for its spread [49], [60]. Since our review of colonial medical and ethnographic papers reveals that no CSWs with levels of sexual promiscuity comparable to those operating in the West existed in forested equatorial areas before organized colonialism (excepting in the coast and in the savannah-forest interface regions frequented by Arab traders) [56], [61]–[63], we assume that it was this absence of CSWs that was keeping syphilis, chancroid, and the other STDs at bay.

In the period 1890–1920, colonization produced generalized social disruption, sex work flourished, and syphilis (and to a lesser extent chancroid and LGV) invaded all these areas [50], [52]–[54], [57]. Except for tertiary and purely serological diagnoses, colonial doctors of this period were not mistaking yaws for syphilis. Most yaws cases are presented in children [52]; unlike syphilis, yaws is not venereal, seldom affects mucosa, and does not cause primary chancres [53], [64]. In addition, syphilis appeared correlated in time and space with other STDs and with presumed sexual promiscuity in a community (e.g, syphilis was frequent in the colonial posts, and absent in the still undisturbed villages around, and its incidence raised in the posts upon arrival of ships, caravans and military contingents [50], [52], [54], [58]).

A common ironical pun was “*Nous leur avons apporté la syphilization*” (“We have brought them syphilization”). GUD invasion accompanied the social disruption that resulted from colonial development of each region [47], [48], [50], [52]. We hypothesize that this promoted sexual transmission of several zoonotic SIVs. Among these zoonotic strains, those arriving to cities, not only could rapidly generate a larger hub of infected people but also, being placed at a major traffic node, would have had more long-term epidemic possibilities. Cities started to grow fast, and riverine traffic intensified only after 1920 [47], [65].

**Figure 1:**
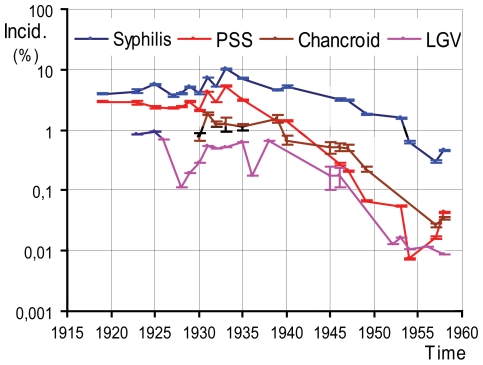
Annual incidences of several GUDs (in %) in Kinshasa. Declines of 1.5–2.5 orders of magnitude happened between the 1920s and the 1950s. (Data compilation in Text S1).

In Kinshasa (then Leopoldville), capital of the DRC (then Belgian Congo), GUD was much more intense in its early growth period, and then declined steadily after the mid 1930s (Figure 1; Text S1).

Starting to grow fast in 1919, the Kinshasa population tripled to about 47,000 by 1929 [47], accompanied by increasing river and railway traffic. Commercial sex work became widespread, not least because of the extremely male-biased (4∶1) sex-ratio [47]. Of 6,000 women living in the eastern part of the city in 1928, only 1,724 were married, 1,600 lived in “illegitimate relationships of more or less duration”, and the remaining (45% of the female population) were presumed to “live mainly on prostitution” [66]; we must stress that only some of these would be pure CSWs; colonial authorities commonly used this derogatory categorization whenever they suspected that women were having multiple sexual partners. By this time, PSS and other GUDs had very high incidences (Figure 1). Before 1919, reports suggest that GUD incidences were probably higher [67]–[69], but archival sources are very incomplete for this period. In 1930–32, large surveys covering most women from the city showed that about 5% had active genital ulcers at the moment of the visit [48].

By 1928, there was a decided colonial response to these medical conditions. Mass surveys, movement restrictions, monitoring of sex workers and treatment of venereal diseases were initiated and were broadened further in scope and technically improved after 1932 [47], [48]. Surveillance and treatments were successful, and after the mid 1930s, GUD incidences declined. During this period, the proportion of syphilis cases representing PSS also declined, from being the majority in the twenties, to only 1–9% in 1949–58 [47], [48], [70], in agreement with epidemiological simulations [45]. With penicillin adding up to old arsenic, bismuth, and sulfonamide treatments after 1947, incident ulcerative syphilis, chancroid, and LGV became residual. PSS cases declined to 40–60 per year in 1949–51, and to 10–25 in 1954–58, these representing incidences of about 1.5–2.5 orders of magnitude lower than those of the period 1919–35 [47], [48], [70] (Figure 1; Text S1).

Similar trends were observed in other African cities [47], [71]–[75], although their timings were not exactly in concordance with those of Kinshasa. For example, in Douala, Cameroon, syphilis represented 13.5% of morbidity in 1923 [59] and only 0.20–0.91% in the period 1935–39 [71]–[73], an amelioration attributed to intensive surveys and treatments [72]. In Brazzaville, syphilis represented 3.0–7.6% of morbidity in the period 1930–34 [74], and only 0.33–1.11% in the period 1953–57 [75]. In the same city, the proportion PSS/syphilis also declined from 84–92% in 1933–34 to 8–34% in 1953–57 [74], [75]. After 1945, GUD incidences became extremely low in urban settings [47], [48], [70], [72], [73], [75].

Although we cannot exclude reporting biases concerning GUDs (or any other diseases) in colonial reports, these biases are likely to be less of an issue for cases detected in the major cities from the 1920s onwards, when health systems became better established. For this reason, we attempt to quantify GUD incidences only for cities, and from 1919 onward, despite having reviewed many other reports beyond these bounds.

Genital herpes (caused by the herpes simplex viruses (HSV), most often by HSV-2) plays a major role in HIV transmission nowadays, but its slow monotonic spread made it to be an important cause of genital ulcers in Africa only after the mid eighties [76]. Accordingly, HSV-2 seroprevalence in Kinshasa, in 1959, was 21% (and 6% in rural Congo), and it took 26 years to attain 60% [76].

In summary, the period 1945–80 is characterized by a low intensity of the four main GUDs in major cities: PSS, chancroid, and LGV became rarer due to the better health systems, and penicillin use; PSS became a small fraction of treated syphilis cases; and genital herpes prevalence was still low. The incidences of the three former GUDs in cities showed peaks up to the mid thirties, when the cities were still small (10,000–50,000 inhabitants), sex ratios were very male-biased, and health systems were incipient.

### City growth does not match in time and in space with the origin of epidemic HIV clades

**Figure 2:**
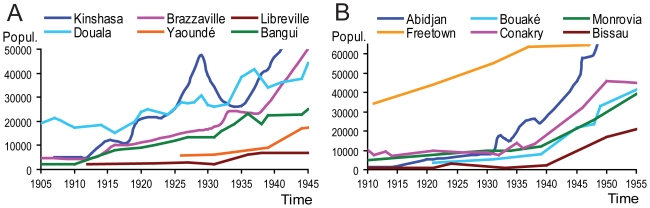
The growth of the most relevant Central African (A) and West African (B) cities. Plotted the evolution of the population of the most relevant cities at or near: (A) chimpanzee and gorilla ranges in Central Africa; (B) sooty mangabey range in West Africa. The supporting references are listed in Text S2.

City growth is a factor to be considered when investigating the emergence of epidemic HIV because a fast growing city potentially receives more SIV-infected migrants per unit time, and can spread the virus among more inhabitants. We examined the curves of population growth of the major Central and West African cities that lie within or near the chimpanzee, gorilla, or sooty mangabey ranges, and that received immigrants from within these ranges (Figure 2). Periods of fast growth span all over the century; growth rates in mid 20^th^ century were among the highest, and involved much higher absolute number of migrant arrivals.

As Figure 2A shows, up to the early thirties, Kinshasa and Douala where clearly the largest cities in the chimpanzee and gorilla ranges. Kinshasa is the recognized early epicenter of HIV-1 group M [2], [29], [77], [78], and Douala was, in early 20^th^ century, by far the largest city of Cameroon, which is most likely the country where the early epicenter of HIV-1 group O developed [15], [17], [18]. The two cities were experiencing high GUD incidences at the time of the TMRCAs of these HIV groups [48], [59] (Text S1). Thus, at the same time, two different epidemic HIV clades emerged in Central Africa in the early 20^th^ century.

Both HIV-2 groups A and B originated most likely in Côte d'Ivoire [9]. Urban development was tardy in this colony; the capital Abidjan only grew fast and surpassed 10,000 inhabitants after 1932 (Figure 2B). GUD levels were high throughout the thirties [79], [80]. It is thus consistent with our hypothesis that the estimates of both HIV-2 groups' TMRCAs fall in this timeframe, a few decades later than HIV-1 groups M and O [6], [31]. However, Freetown was already of considerable size before Abidjan, Conakry grew strongly after 1933 (Figure 2B), to our knowledge these cities did not differ much from Ivorian cities in their GUD levels, and no epidemic HIV-2 seems to have emerged in them. This motivated us to investigate other factors that may explain why HIV groups emerged only in particular cities.

City growth is not restricted in time with the emergence of HIV groups. Cities continued to grow, well after the origin of the epidemic HIVs. While there is some spatial coincidence in Central Africa between city size and origin of HIV-1 groups, this is not the case for West Africa and the origin of the HIV-2 groups.

### Male circumcision patterns in Central and West Africa show some correlation with HIV emergence

Male circumcision reduces the risk of HIV acquisition in men [34], [38], [81], [82], and HIV/AIDS prevalence correlates inversely with the level of male circumcision in Africa [83]–[86]. Recent randomised trials of male circumcision demonstrated a marked reduction in male susceptibility to HIV infection [87], [88]. We hypothesized that circumcision levels in cities might also have influenced the chances of HIV emergence from zoonotic SIV transmissions.

We studied the geographical distribution of circumcision patterns in Central and West Africa both today and at the time of the HIV groups' TMRCAs, to evaluate if it correlated spatially and in time with HIV emergence. We reviewed all the Demographic and Health Surveys (DHS) [89], pertaining to the relevant countries, and additional studies [90], [91], which reported circumcision levels. We found that circumcision is nowadays nearly universal in the countries of the chimpanzee and sooty mangabey ranges, except for Rwanda, Burundi, Uganda, and Tanzania (Table S1).

This near universality contrasts with what can be inferred from Murdock's Ethnographic Atlas [92], [93]. Also due to other inconsistencies in this Atlas, we decided to gather primary ethnographic papers, putting more focus on the period of HIV emergence (1900–1940) (see Materials and Methods, section circumcision study). Our survey extensively expanded upon currently available circumcision information for the relevant ethnic groups [94], and permits a detailed study of the geographical distribution of male circumcision during colonial times in the areas of chimpanzees and sooty mangabeys (Table S2).

We found that, in the early 20^th^ century, circumcision patterns in Central and West Africa exhibited much stronger regional differences than nowadays. Peoples of the Adamawa-Ubangi linguistic cluster (occupying most CAR and northern DRC), and many Bantu peoples of the Orientale and Équateur provinces of DRC, adopted it in late 19^th^–early 20^th^ century [95]. In Rwanda and Burundi, circumcision was not practiced, a pattern that persists today [95], [96] (Text S2). In West Africa, most ethnic groups were circumcised, with some exceptions (e.g., the Akan peoples from eastern Côte d'Ivoire and Ghana, and many Gur peoples from northeastern Côte d'Ivoire, Burkina-Faso, and Ghana) (Text S2).

**Figure 3:**
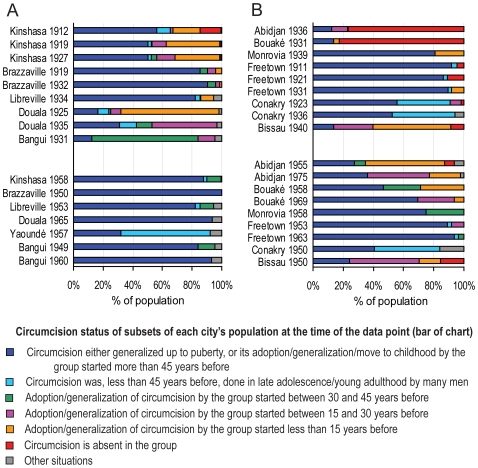
Male circumcision patterns in Central African (A) and West African (B) cities. The charts show, for each city, and at the referred time, the proportional distribution of the male population by “circumcision classes” which are directly derived from the ethnographic literature and do not depend on additional assumptions. Each bar is based on either: i) a published census or survey partitioning by ethnicity; ii) assumption of the same ethnic distribution as in a neighboring time point for which there is a census or survey; iii) published numbers for some ethnic groups, and estimates for some relevant others. The proportions of red and orange in each bar indicate the proportions of the population belonging to groups which, respectively had not adopted circumcision by the time of the data point (red), or had adopted it, or started to generalize it from a situation in which it is described as far from general in the ethnographic literature, less than 15 years before the time of the data point (orange). So, higher proportions of red and orange (and, to a lesser extent, pink) mean lower circumcision frequencies. See supporting information in Text S2, and supporting calculations in Dataset S1.

**Figure 4:**
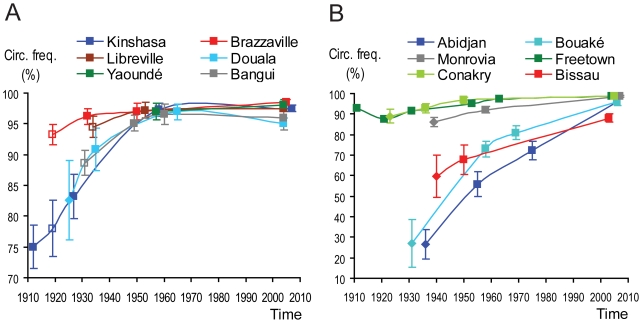
Estimates of male circumcision frequencies in Central African (A) and West African (B) cities. The charts show, for each city, and at the referred time, the upper and lower estimates of male circumcision frequency. The cities and times of estimates are the same that appear in the bars of Figure 3. Each estimate is based on either: i) a published census or survey partitioning by ethnicity (filled squares); ii) assumption of the same ethnic distribution as in a neighboring time point for which there is a census or survey (shallow squares); iii) published numbers for some ethnic groups, and estimates for some relevant others (lozenges); iv) present time estimates for each city are assumed to be similar to the national prevalences measured by the DHS, because the latter are above 95% for nearly all relevant countries, and this, considering the current high levels of ethnic mixing seen in African major cities, leaves little room for a major city to differ from the national average. Except for the situation iv) above, circumcision frequencies are estimated based on the ethnographic information about the circumcision practices of each group, according to an algorithm described in Text S2, and the supporting calculations are implemented in Dataset S1.

For the main cities of the relevant areas, we collected demographic surveys at several points in time which discriminated the urban population by ethnic group. To each ethnic group present in a city, at a given time, we assigned a “circumcision class” (e.g., generalized at puberty, absent, etc), based on the information provided in ethnographic sources, and we calculated upper and lower estimates of frequency of circumcision in male adults (see Materials and Methods, section Circumcision prevalence survey, Text S2, and Dataset S1). This permitted us to calculate, for each city, and at a given time, the distribution of its male population by the defined circumcision classes, and lower and upper estimates of circumcision frequency. The results are displayed in Figures 3 and 4.

Among the three Central African cities which were clearly outstanding in size before the 1930s (Kinshasa, Douala, and Brazzaville (Figure 2A)) the first two (the proposed epicenters of HIV-1 groups M and O) had lower circumcision rates (Figures 3A and 4A). During the period 1910–35, Libreville, Bangui, and Yaoundé may have had lower circumcision rates than after World War II, but we could not ascertain this by lack of adequate tables of ethnic composition. We did not include Rwandese and Burundian cities in our study, because they were very small up to mid 20^th^ century [65], and the cattle raising tradition of these countries makes bushmeat practice uncommon [93], [97], [98].

Among the four West African cities that clearly stood out in size before World War II (Abidjan, Freetown, Monrovia, and Conakry (Figure 2B)) the first had a much lower circumcision rate (Figures 3B and 4B). Both HIV-2 epidemic groups (A and B) appear to have originated in Côte d'Ivoire [9], as well as the dead-end/rare infections of groups G and H [7]–[9]. Therefore, the match between lack of circumcision in cities and HIV emergence appears to be stronger for HIV-2 than for HIV-1. Although Côte d'Ivoire contains only about 5% of the sooty mangabey range [9], [13], it is the country of origin of half of the identified HIV-2 strains, and Ivorian cities, such as Abidjan and Bouaké, had much lower circumcision rates up to World War II than the other West African cities (Figures 3B and 4B). Our findings of significant differences between the cities are robust and independent of the assumptions we introduced to estimate circumcision rates (expressed in Figure 4); they are clearly demonstrated in Figure 3 (which does not work with circumcision rate estimates).

To further substantiate these observations, we gathered tables of surgical operations discriminating circumcisions to treat phimosis and paraphimosis in major cities [71], [74], [99]–[106]. Ethnic groups performing universal circumcision almost always did it either at puberty with rituals, or in early childhood without rituals (Text S2; Dataset S1); in the first case, we assume parents would wait for puberty to circumcise the boy within the tribal rituals, even if he had phimosis; in the second case, a boy could have phimosis only during the first years of life. Therefore, ethnic groups performing universal circumcision should contribute little to the statistics of circumcisions for phimosis made in the colonial health system. These statistics should include mainly males from groups not performing universal circumcision. Therefore, we assume that high numbers of such recorded operations in a city reflect a relatively high proportion of uncircumcised adults living there. We display the results in Table 2.

**Table 2:** Incidences of phimosis and paraphimosis in several African cities.

Country	City/division	Years	# of cases	Annual incid.	Refs.
Belgian Congo	Kinshasa	1907, 1910–12	74	0.685%	[99]–[102]
Belgian Congo	Kinshasa	1926	46	0.357%	[103]
Belgian Congo	Kinshasa, Matadi, Boma^a^	1930–31	245	0.395%	[104]
French Congo	Brazzaville	1930–34	89	0.265%	[74]
Cameroon	Douala/Wouri^b^	1932, 1935	313	0.635%	[71]
Mali	Bamako	1937	4	0.045%	[105]
Senegal	Saint Louis	1937	6	0.040%	[106]

Presented the joint annual incidences of phimosis and paraphimosis in the males of several African cities, as reported in the medical references listed in the last column. Demographic data for each city is in [65] and in the references listed in Text S2.

^**a**^The incidence is calculated over the joint adult male population of the three cities; about 3/5 of the operations were in Kinshasa.

^**b**^Most people from Wouri division were in Douala city [71].

The low incidences of phimosis in Mali and Senegal are explained by the Islamic practice of circumcision in childhood. The phimosis data support the findings of our ethnographic study that circumcision was far from general in Central Africa in 1910–35, and of lower rates in Kinshasa and Douala than in Brazzaville (Figures 3A and 4A; Text S2; Dataset S1). Table 2 presents all the phimosis statistics we found that *referred to a city*; in addition to these, we collected many dozens of other phimosis statistics *at the country level*. They tend to corroborate the between country differences in circumcision levels that we obtained through the ethnographic approach (data not shown).

In our ethnographic study, we seized the opportunity to survey not only patterns of male circumcision, but also patterns of primate hunting. We present the results of this survey in Table S3. Hunting of chimpanzees and gorillas was very widespread among the ethnic groups of Central Africa, as was the hunting of monkeys in Central and West Africa (Table S3). Furthermore, SIVcpz and SIVsmm have a very wide geographical presence across the range of their primate hosts [2], [9], [107]. Therefore, differences in hunting practices are unlikely to be the major factor explaining why HIV strains emerged only in some countries.

In conclusion, male circumcision rates in Central and West Africa were generally lower, and showed more pronounced regional differences in early 20^th^ century than nowadays. Low circumcision levels in cities also appear to match early HIV epicenters and this is more evident for HIV-2 in West Africa than for HIV-1 in Central Africa.

### Simulating the early spread of HIV in Kinshasa

Finally, we used computer simulations to verify that the time window for the emergence of epidemic/pandemic HIV strains indeed offered uniquely favorable conditions for the heterosexual spread of the virus. Because the window of opportunity may have involved simultaneous changes in several factors (population size, sex ratio, sexual promiscuity, GUD and circumcision prevalence), we also wanted to evaluate the individual contribution of each factor to successful epidemic emergence.

We focused on the origin of HIV-1 group M in Kinshasa for which we were able to collect the most complete historical data. Our simulations were parameterized to follow the recorded population size and structure of Kinshasa at several relevant time points, partly based on the availability of detailed population and medical records (Table 3). The years 1919 and 1929 were chosen from the time interval estimated for the origin of HIV-1 group M [27]–[29]; both time points were characterized by rampant GUD epidemics, highly male-biased sex ratio and lower levels of circumcision than today. The year 1958 was chosen as a time point beyond the window defined by our phylogenetic dating study; although the city population had considerably expanded, GUD infections were generally under control and circumcision was almost universal. Finally, we have explored a “pre-colonial village” scenario to reflect a large settlement in the region before colonization, characterized by a healthy population structure and the absence of GUD infections and sex work.

**Table 3:** Scenario-specific parameters of the simulations.

Parameter	Pre-colonial village	Kinshasa 1919	Kinshasa 1929	Kinshasa 1958
Number of women	500	3,265	10,081	69,159
Number of men	500	8,798	31,817	93,064
Number of married couples	450	947	2,923	48,411
% of women “*femmes libres*”	0	60	60	10
% of men circumcised	0	70	80	95
Genital ulcer frequency (%) in:				
Commercial sex workers (CSWs)	-	15	10	5
*Femmes libres*	0	7.5	5^a^	0.5
Other women	0	3	2	0.3
Men	0	1.5	1	0.3

The parameters are based on archival historical data and colonial medicine articles ([48], [66], [70], [104]; Text S1; Text S2).

^**a**^A genital ulcer frequency of 5% in *femmes libres* in 1929 is supported by two different venereal control surveys made in 1930 and 1932, involving 953 and 1,202 women (mostly *femmes libres*) respectively, which showed this frequency to be 4.7% in 1930 and 4.6% in 1932 [48]. The venereal situation was probably even worse in the decade preceding 1929–32 [48], [104], and reports from the years around 1910 [67]–[69] suggest incidences higher than in the period 1929–32 (see Text S1).

Our simulations followed the early spread of the epidemic from the first zoonotic SIV infection over a dynamic network of sexual contacts, which was parameterized according to recent surveys [108]–[114] (see Materials and Methods and Table 4). Transmission parameters were modeled after current HIV data, adopting the estimated relative effects of modifiers (e.g. GUD infection) [33], [34], [38], [81], [82], [115], [116], but assuming a lower baseline transmission probability for ancestral HIV. The modifier effects of GUD infections and circumcision were implemented in the transmission process. We also assumed that transmission of these early strains (not yet adapted to humans) was limited to acute infection, as has been observed for recent cross-species SIV transmissions [21]. The network of contacts was parameterized according to recent studies [108]–[114], with the sexual promiscuity of “*femmes libres*” (an expression used by colonial writers to refer to unmarried/unmated women to whom a high rate of sexual partner change was attributed [13], [47], [48], [70]) adjusted according to the sex ratio (see Materials and Methods and Table 4 for all scenario-independent parameters). We provide the code of our simulations in Text S3.

**Table 4:** Scenario-independent parameters of the simulations.

Parameter (notation)	Default value/Formula [references]	Other values tested [references]
***Network parameters*** a		
Number of non-spousal partners per year^b^		
Single women (N_SW_)	1.5 [108]	
Married women (N_MW_)	0.2 (estimated from data in [109])	
Single men (N_SM_)	3 [108]	4.5^c^
Duration of short links^b^ (weeks) (D)	52 (in [109] as reported by women)	26^c^ (in [109] as reported by men)
Number of sex acts per week^b^		
Stable (spousal) link	2 [110], [111]	
Short link	0.24 (estimated from data in [109])	2^d^
CSW visits per man per year^b^ (C)	2 [109], [112]	0^e^, 8^f^
Sex acts per CSW per year (S_C_)	600 [113]	150^e^ (estimated from data in [109], [114])
***Derived parameters***		
Probability of short link breakup per week (p_b_)	1/D	
Probability of short link formation		
Single women	N_SW_/(D+52)	
Married women	N_SM_/(D+52)	
Single men (p_f,SM_)	N_SM_/(D+52)	
Married men^g^	p_f,SM_–p_b_	
Number of CSW	(Number of men)*C/S_C_	
Probability of CSW visit per man per week	C/52	
***HIV parameters***		
Duration of acute infection (weeks)	12 [115]	
Transmission multiplier for acute infection	10	4, 26 (estimate for modern HIV-1 [115])
Maximum per-act transmission probability	0.9	0.43 (equal to highest observed heterosexual rate [34])
Per-act transmission probabilities^h^		
♂→♀ and ♀→♂(C)	0.001 [116]	
♀→♂(NC)	0.0025 [81], [82], [116]	0.01 [34], [38], [116]
♂→♀(GU)	0.07 [33]	
♂(GU)→♀ and ♀(GU) →♂(C)	0.04 [34]	
♀ (GU) →♂(NC)	0.43 [34]	
♀ →♂(GU)	0.023	
♂(GU)→♀(GU) and ♀(GU)→♂(GU)	0.43	
***GUD parameters***		
Duration of GU episodes (weeks)	10 [45], [46], [49]	

^**a**^The studies in [108]–[110], [112], [114] surveyed several African cities: we used the estimates provided for Yaoundé, which was the location nearest to Kinshasa.

^**b**^These parameters determined means for random distributions, rather than fixed values.

^**c**^Both combinations of 3 partners per year with 52-week short links and 4.5 partners per year with 26-week short links yielded a concurrency index around 1.5, consistent with [108].

^**d**^Employing the same value as for stable links.

^**e**^The line between CSWs and *femmes libres* is blurred. We therefore ran also simulations without professional CSWs, and with CSWs with intermediate sexual promiscuity.

^**f**^There were 14 CSWs per 1,000 men in Yaoundé according to [114]. With 600 acts per CSW per year, this would yield about 8 CSW visits per man per year.

^**g**^This formula implies the same number of concurrent links (including the spousal link) but fewer annual partners for married compared with single men.

^**h**^(GU) indicates a partner with an active genital ulcer; (C) indicates a circumcised man; (NC) indicates a non-circumcised man.

**Figure 5:**
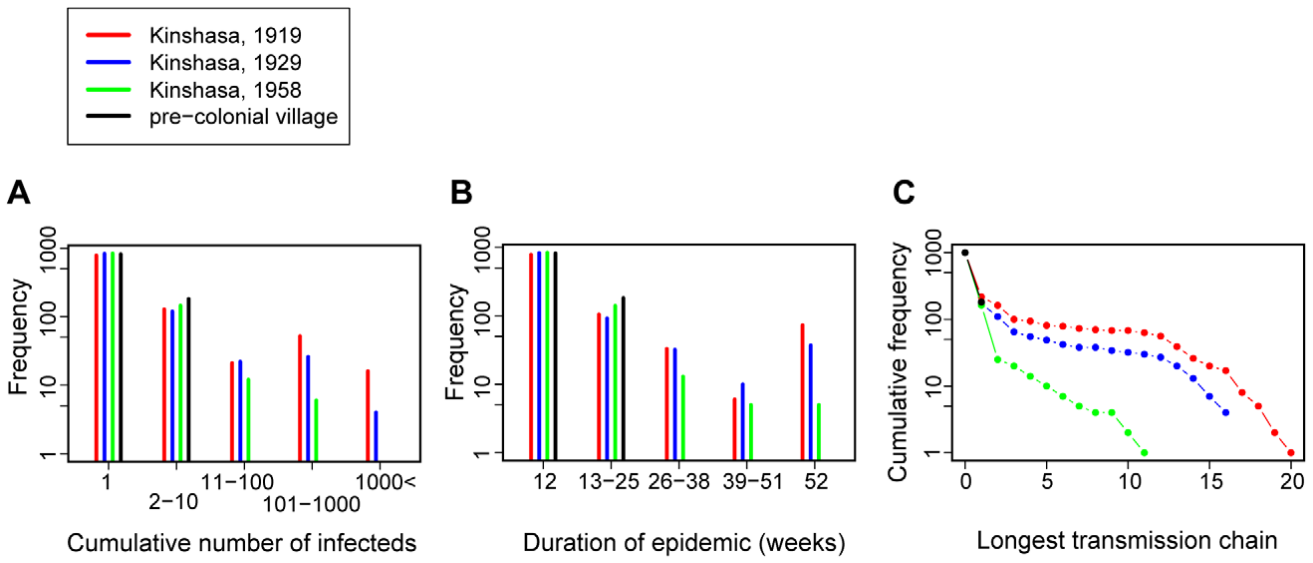
Comparison of early spread of HIV in simulated historical scenarios. The graphs depict frequency distributions of the total number of infections per simulation (A), the duration of the epidemic (B) and the longest chain of transmission (C) from 1,000 simulations of Kinshasa in 1919 (red dots and bars), 1929 (blue dots and bars) and 1958 (green dots and bars), and a pre-colonial village (black dots and bars). The duration of an epidemic was defined as the time until the resolution of the last acute infection: its lower bound was defined by the length of acute infection in patient zero (12 weeks), its upper bound by the length of the simulations (52 weeks). The longest transmission chain was defined as the number of individuals in the longest chain of subsequent transmissions in each simulation. All frequencies (number of observations) are plotted on a log scale.

We defined several markers to characterize the efficiency of epidemic spread in the simulations (Figure 5). Per simulation, we determined the total number of infections (Figure 5A) and the duration of an epidemic (Figure 5B), which characterize the extent of the first outbreak of infections and the ability of the virus to persist in the population even in its initial ill-adapted form (with reduced transmission efficiency compared with modern HIV). Long-term establishment (epidemic emergence) of HIV probably also depended on rapid initial adaptation to the new human host species. The capacity for this adaptation is also determined partly by the total number of human hosts and the duration of the epidemic (the age of the oldest lineage). However, an additional important determinant of adaptation is the length of the longest transmission chain (Figure 5C), i.e. the number of “serial passages” in human hosts.

In all three quantifiers of epidemic emergence, the performance of the historical scenarios followed the same pattern: Kinshasa 1919> Kinshasa 1929≫ Kinshasa 1958≫ pre-colonial village (Figure 5). E.g. compared with the 1958 scenario, the 1929 scenario had 5-fold, the 1919 scenario 11-fold higher chance to generate at least 100 infections (6, 30 and 68 times out of 1,000 simulations); the pre-colonial village scenario never generated more than two infections. The probability of the ill-adapted virus to persist until the end of the simulated year was also dramatically higher for the 1919 and 1929 scenarios compared with the pre- and post-origin scenarios (73 and 37 vs. 5 and 0 times out of 1,000 simulations), as was the probability of generating a transmission chain of at least length five (81 and 49 vs. 10 and 0 times out of 1,000 simulations; see Table S4 for more detailed simulation outcomes). We thus found that the scenarios dated around the origin of HIV-1 group M (1919 and 1929) were indeed much more permissive for the heterosexual spread of emergent HIV compared with scenarios dated either before or after the estimated origin. This result proved to be robust with respect to varying a number of parameters in the model (Table S4). Note also that even in the most permissive scenarios, the initial zoonotic infection was a dead end in more than 50% of the simulation runs. Furthermore, the more permissive 1919 and 1929 scenarios yielded a bimodal distribution of outcomes indicating the effect of early stochastic events: after the first few transmissions, the epidemics that happened to reach the highly connected core of the sexual network can spread extensively; those that fail to do so, are likely to die out quickly.

**Figure 6:**
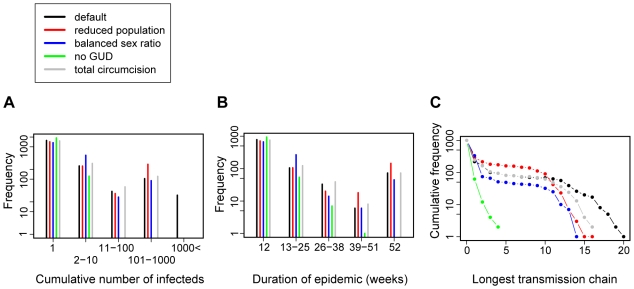
The effect of selected factors on the simulated early spread of HIV for Kinshasa 1919. The graphs depict frequency distributions of the total number of infections (A), the duration of the epidemic (B) and the longest chain of transmission (C) from 1,000 simulations of Kinshasa with default parameters (black dots and bars), 10-fold reduced population size (red dots and bars), balanced sex ratio (blue dots and bars), no GUD (green dots and bars) and universal circumcision (gray dots and bars). The duration of an epidemic was defined as the time until the resolution of the last acute infection: its lower bound was defined by the length of acute infection in patient zero (12 weeks), its upper bound by the length of the simulations (52 weeks). The longest transmission chain was defined as the number of individuals in the longest chain of subsequent transmissions in each simulation. All frequencies (number of observations) are plotted on a log scale.

While the resistance of the pre-colonial village to HIV emergence is not surprising, the dramatic decrease in permissiveness between 1929 and 1958, in spite of continued explosive population growth, demands further explanation. Furthermore, the 1919 scenario proved to be consistently more permissive than the 1929 scenario, in spite of considerable population growth over the decade. To identify the key factor(s) behind the observed differences, we explored systematically the effect of removing or reducing several factors that have been implicated in the emergence of HIV. Based on the most permissive 1919 scenario, we tested 10-fold reduced population size, balanced sex ratio (with 90% of the sexually active population in stable relationship), absence of GUD infections and universal circumcision. The removal of GUD infections proved to have by far the most dramatic effect (Figure 6). Remarkably, both a strongly reduced population size (∼1,200 sexually active individuals) and universal circumcision had a much weaker effect on the spread of ill-adapted HIV in the simulations. We also explored all combinations of these mitigating factors and found a consistently dominant effect of GUD prevalence (Table S5). We thus conclude that the period around the estimated origin of HIV-1 group M was uniquely permissive for the emergence of the virus by heterosexual transmission, and that the unprecedented GUD epidemics of the time were the main contributor to this high permissiveness.

## Discussion

We present multiple lines of evidence favorable to the hypothesis of rampant GUD epidemics having played a key role in the origin of the major HIV strains.

In agreement with earlier studies our molecular dating confirmed that all major epidemic HIV lineages were transmitted to our species in a narrow time frame. We dated the divergence of the HIV-1 groups M and O from their closest related SIVs using a different, but complementary approach compared to Wertheim and Worobey (2009) [31]. Whereas Wertheim and Worobey (2009) aimed at estimating the TMRCA of SIV in chimpanzees and sooty mangabeys [31], we focused on obtaining an upper bound on the cross-species transmission from the chimpanzee species. To this purpose, we focused on separate *pol* data sets for HIV-1 group M/SIVcpz and HIV-1 group O/SIVgor/SIVcpz. Because the relatively conserved *pol* gene does not contain sufficient temporal signal (which may explain the relatively low rate and old TMRCAs of Wertheim and Worobey (2009) for a similar *pol* data set), we calibrated the phylogenies using the group M and group O TMRCAs respectively. Therefore, we essentially extrapolated on the rate of HIV-1 evolution, but also Wertheim and Worobey (2009) [31] noted that the SIVcpz sequences could not be used on their own for meaningful date estimates. We obtained a relatively narrow timeframe for the interspecies transmissions, defined, for each HIV group, by the period between the split from the closest SIV and the intragroup TMRCA. Moreover, interspecies transmission and possible adaptation to humans probably happened close to the latter date, which would restrict the timeframe even further.

Thus, we looked for factors to explain why emergence of HIV is temporally and spatially restricted to the era and areas observed. Our review of the co-factors of sexual transmission indicated GUD as paramount and lack of male circumcision of secondary importance. GUD provides a portal of entry and attracts cells carrying CCR5, the co-receptor most used by HIVs and SIVs upon sexual transmission. In addition, GUD and especially syphilis induces a potent inflammatory response, and tumor-necrosis-factor (TNF)-α production [117], which is a major enhancer of HIV replication [118]. Genital ulceration and inflammation in humans contributes strongly to the odds of acquisition of more than one HIV-1 viral variant [119]; and transmission of multiple viral variants was shown to contribute to adaptation to a different host species in experimental infection of chimpanzees with HIV-1 [120], [121] and rhesus macaques with SIVsmm [122]. These processes suggest that GUD may contribute to SIV adaptation in ways beyond increased transmissibility.

Most theories for the origin of HIVs depend on a specific mechanism to facilitate the first few serial transmissions of the virus in humans, and largely limit the problem to initial adaptation [4], [5], [13], [23]. However, the emergence of an epidemic might also have depended on the conditions for large scale spread in the general population by the conventional route, i.e. by heterosexual transmission. Without favorable conditions for further spread, even a virus that passed initial adaptation might quickly go extinct. While we cannot exclude that the initial adaptation of HIVs depended on specific transmission routes (e.g. parenteral transmission), we investigated the possibility that epidemic emergence may have depended on large population centers riddled with sexual promiscuity and GUD. Bearing in mind that few cities in Central and West Africa were well developed during the peak GUD period (Figure 2), our hypothesis may explain why few well-adapted strains emerged; and it may not be coincidence that exactly two HIV-1 groups emerged in Central Africa, at a time when two cities (Kinshasa and Douala) stood out in the region (Figure 2A). Therefore we focused on the co-factors sexual promiscuity, GUD, and lack of circumcision in cities. Our review of the colonial medical literature established that GUD, particularly syphilis, chancroid, and LGV, peaked in the relevant cities, in the period 1910–35, with incidences 1.5–2.5 orders of magnitude higher than in mid 20^th^ century, coinciding in time with the narrow timeframe of the emergence of epidemic HIV groups.

Our computer simulations of detailed historical scenarios for Kinshasa confirmed that the period around the origin of HIV-1 group M in the city was uniquely permissive for the emergence of an epidemic by heterosexual transmission. While exact probabilities of HIV emergence cannot be computed (e.g. we have no information on the initial infectivity of a novel zoonotic HIV), our semi-quantitative approach could robustly predict an increased relative probability associated with this time period. Furthermore, our simulations suggested that the peak in GUD prevalence was the most important contributor to chains of transmission of ill-adapted HIV. A related important result of the simulations is the inability of zoonotic HIV to generate epidemics in the pre-colonial village scenario (characterized by the absence of GUD and CSWs), which explains the long standing absence of HIV epidemics in the pre-colonial environments. According to these results, the window of high permissivity for epidemic HIV emergence was open by the spread of GUD infections due to the organized colonization of the relevant African areas, and probably closed by the aggressive treatment campaigns against GUDs from the mid thirties. Therefore, we predict that newly emerging HIV groups will have a less dramatic spread if GUD remains under control. Remarkably, the direct effect of population size and circumcision proved to be relatively small, although their effect is recognized and they may have acted indirectly.

In the simulations, the probabilities for sexual link formation and breakup were the same for all individuals of a class (e.g. single men, married women, etc). For the sake of simplicity, we did not implement a “small world network” [123], [124]. However, the role of highly-connected “hubs” was explored by adding highly promiscuous CSWs with various settings to the simulations. Furthermore, increasing the proportion of highly-connected individuals in the population by employing a power-law distribution for the number of partners would only have enhanced heterosexual transmission even further.

Simulation models have been used before to estimate the contribution of sexually transmitted infections/GUDs to the current heterosexual spread of HIV [37], [125]. However, our study is the first to attempt a semi-quantitative assessment of the role of GUDs in the origin of the epidemics. Our model was tailored to focus on the early spread of HIV, which allowed for a simplified model structure.

We hypothesized that differences in male circumcision levels between cities may help to explain why HIV zoonotic strains emerged only in particular countries. Our extensive survey revealed circumcision patterns that were historically low in the putative centers of HIV emergence (Kinshasa, Douala, and Abidjan). Our simulations showed only a moderate direct effect of circumcision in the probability of generating long chains of transmission. However, lack of circumcision also favors GUD transmission [49], [126], [127], and low circumcision levels might have correlated with high GUD incidence. The prevalence of circumcision might thus have affected HIV emergence indirectly through its effect on GUD incidence. Lack of circumcision may have been more important for HIV-2 emergence, because epidemic HIV-2 groups emerged *only* in Côte d'Ivoire, a country which cities had *much lower* circumcision rates than the others of the region in the critical period (Figure 3B).

Independently of the regional differences encountered, our finding of a very widespread trend of adoptions of circumcision, in early 20^th^ century, by ethnic groups previously not practicing it, and the resulting temporal increase of circumcision rates in most relevant countries, is a solid result. It explains, as far as we know for the first time, the discrepancy between modern levels of circumcision, as showed by the Demographic and Health Surveys (DHS) [89], and the levels inferred from the Ethnographic Atlas [92], [93].

Independent of whether lack of circumcision was important to HIV adaptation, its geographical distribution may have determined to a large extent, which secondary foci developed in the decades after early emergence. Our finding of a relatively low circumcision rate in Guinea-Bissau may reinvigorate the debate about why this country became an early important focus of HIV-2 group A. In this regard it is important to note that some of the earliest transnational jumps of pandemic HIV-1 happened to countries where circumcision is uncommon: Haiti [128], Rwanda, Burundi, Zambia [83]–[86], [89], and Thailand [85].

Our simulations suggest that city size *per se* was not an important factor for initial HIV transmission. Therefore, we cannot rule out that the first transmissions (and possible initial adaptation of the virus) occurred in smaller settlements such as Bangui, Yaoundé, Kribi or Brazzaville. However, the larger size of Kinshasa and Douala in that period may have been important for, at least, three reasons. First, a larger city attracts more immigrants per unit time, and hence potentially more SIV infections. Second, their larger size reflected early industrialization associated with start-up infrastructure projects (fluvial and sea harbors, railways), and this led to hasty recruitment of young male labor force, and thus to a extremely male-biased sex ratio, favoring commercial sex work and GUD. In the 1920s and 1930s, industry, public works, and business in general, were more advanced in Kinshasa and Douala than in the other Central African cities. Accordingly, sex work was “by far more flourishing” in Kinshasa than in Brazzaville [129]. Douala was also a major center of sex work and GUD [59], [71], [130], [131]. In West Africa, sex work was widespread in Abidjan [79], [132], whereas it only “existed on a small scale” in Monrovia [133]. Thus, high GUD prevalence might have depended indirectly on population size.

Third, while initial bursts of SIV spread, and resulting adaptation, might have happened in small settlements, further spread of the epidemic was probably centered on cities with large populations. Large cities were at the center of star-like traffic networks, connecting them to nearby settlements, and allowing for quick transfer of the virus from a local initial outbreak. Furthermore, outbreaks in small settlements might quickly become self-limiting by exhausting the supply of susceptible individuals, and sustained epidemics probably depended on the early transmission of the virus to a large center with fast replenishment of susceptible individuals to maintain the epidemic. Thus, major, well-connected centers, such as Kinshasa and Douala (which were better served by railway and fluvial connections, and had far more traffic than the other cities), may have acted as an “attractor” and a “hub” for HIV epidemics. Although these ideas were not explicitly modeled in this study, they may help to understand why exactly two HIV-1 strains evolved and spread considerably in Central Africa, and perhaps may give clues on the origin of the subtypes.

Our proposal that Kinshasa, Douala, and Abidjan constituted the initial hubs of the epidemic HIV groups can also explain the following historical facts: 1) the presence of already diversified HIV-1 group M in Kinshasa in 1959–60, as evidenced by two seropositive samples (a subtype B/D and a subtype A) in only a few hundred stored blood and tissue samples available for screening [29], [134]; 2) the serologically confirmed evidence that HIV-1 group O was present in Douala's communities of sex workers by 1962 [13]; 3) the widespread presence of HIV-2 in separated locations in Côte d'Ivoire and in Guinea-Bissau (a country 1,000 km away) in the early sixties [13]; this fact is better understood assuming that HIV-2 had expanded in the previous decades in a major, internationally connected, Ivorian city.

Our hypothesis satisfies both temporal and spatial coincidence between the factors which we invoke and the emergence of a pathogen. Such coincidence has previously been considered evidence that the factors are causally implicated [39], including by authors drafting hypotheses on the origins of HIV [3]–[5], [13], [23], [24]. It also offers a conceptual simplicity because it proposes as causal factors for SIV adaptation to humans and initial spread the very same factors that most promote the continued spread of HIV nowadays: promiscuous sex, particularly involving sex workers, GUD, and possibly lack of circumcision. However, we are aware that the evidence we provide does not rule out the possibility of other processes having contributed to HIV emergence and/or adaptation. For example, parenteral transmission might also have contributed to the initial adaptation and/or initial spread of HIV (as seems to have been the case for SIVmac [135]), or to further epidemic expansion. What we claim is that this is not necessary to explain the spatial and temporal patterns of HIV emergence, while high GUD incidence seems to have been the key determinant.

In this study, we narrowed down the origin of the epidemic HIV clades (HIV-1 groups M and O, HIV-2 groups A and B) to the first half of the 20^th^ century, using phylogenetic molecular clock calculations. Our colonial archival literature survey shows that GUD epidemics peaked in cities in their early phases of development, providing a better coincidence with this narrow time frame than the driving factors proposed by other theories. Ethnographic literature illustrates that circumcision frequencies were historically considerably lower, and spatially more variable, than they are currently; in particular for HIV-2, low circumcision prevalence in cities indeed showed a geographical match with emerging HIV epicenters. Through epidemiological modeling we could simulate that early ill-adapted HIV could generate long chains of transmission only during a period of high GUD intensity. The effects of circumcision and city size were more likely indirect, through their capacity to enhance GUD intensity and allowing the initial hub of infections to potentially reach a threshold, and to spawn secondary foci. We conclude that intense GUD in nascent cities was probably the main factor that permitted zoonotic SIV to emerge as epidemic HIV, possibly in association with low circumcision rates.

We hope our hypothesis will increase awareness of the dangers posed by GUD in promoting transfer of SIV, STLV, and possibly other sexually transmitted viruses, to our species. These observations recommend close monitoring and treatment of GUD in Africa, and raise concern over the currently high prevalence of HSV-2 associated genital ulcers. We also underscore the importance of male circumcision in the prevention of novel HIV strain emergence.

## Materials and Methods

### Phylogenetic dating

SIVcpz–HIV-1 group M and SIVgor–HIV-1 group O divergence dates were estimated using a Bayesian relaxed clock analysis implemented in BEAST [40]. For SIVcpz–HIV-1 group M, we analyzed the partial *pol* gene sequences (nucleotide 3887 to 4775 according to the HXB2 numbering) recently obtained from chimpanzees [2], three additional SIVcpz lineages (X52154, AY169968 and AF382828), and HIV-1 group M sequences previously analyzed by Lemey et al. (2005) [136]. For SIVgor–HIV-1 group O, we analyzed partial *pol* gene sequences (nucleotide 4230 to 4775 according to the HXB2 numbering) from three SIVgor [1], two SIVcpz (U42720 and X52154), and HIV-1 group O sequences previously analyzed by Lemey et al. (2004) [30]. We applied a general time reversible model with gamma-distributed rate variation among sites combined with an uncorrelated lognormal relaxed clock model and a piecewise constant population size model [40]. These conserved gene regions in the HIV genome are less subject to substitution saturation, but contain poor temporal signal [30]. Therefore, we used a normal prior distribution on the TMRCA for both HIV-1 group M (1931±8 years) and HIV-1 group O (1915±17 years), based on published dates and their uncertainty [28], [30], to calibrate the molecular clock. Posterior distributions were obtained using Markov chain Monte Carlo (MCMC) analysys and MCMC runs were investigated using Tracer (http://tree.bio.ed.ac.uk/software/tracer/ ).

### GUD incidences survey

We compiled STD and GUD incidence data for all the countries at or near the ranges of chimpanzees and sooty mangabeys through a survey of the relevant literature, of the period 1890–1960, from the following sources:

1) Official colonial reports about health, or containing sections about health, and covering also demographic issues. A) For the Belgian Congo, collected in the Afrika Archief, Federale Overheidsdients–Buitenlandse Zaken, Buitenlandse Handel en Ontwikkelingssamenwerking (FO-BZBHO) (Ministry of Foreign Affairs), Brussels; B) For French Equatorial Africa (AEF), and French West Africa (AOF), collected in the Centre des Archives d'Outre-Mer (CAOM), Aix-en-Provence (France), and in the Institut de Médecine Tropicale du Service de Santé des Armées (IMTSSA), Marseille (France).

2) We complemented this information with articles in the main colonial and tropical medicine journals (Ann Méd Pharm Coloniales, Ann Hyg Méd Coloniales, Ann Soc Belge Méd Trop, Bull Soc Pathol Exotique, West African Med J, and others), and with books on the subject.

We did a complete survey of the GUD incidences in Kinshasa: supporting data and methods are described in Text S1.

We obtained the relevant demographic data about the relevant cities from articles, books, and archival reports (references in Text S2).

### Circumcision prevalence survey

We obtained circumcision information on ethnic groups from the Revised Ethnographic Atlas (Gray (1999) [93], which is based on Murdock (1967) [92]). We considered all relevant ethnic groups in the Ethnologue database [94]. We collected primary ethnographic articles and books to complement the circumcision information provided by the Atlas, thus covering all important ethnic groups, and almost all groups with more than 20,000 people, of the areas at or near chimpanzee and sooty mangabey ranges. Most observations were from the period 1880–1940 (both those on which the Atlas is based, and those of our ethnographic collection). For each ethnic group, we gathered information on male circumcision practice, including generality, approximate time of adoption (if adopted recently), associated initiations and rituals if any, and age of circumcision. We collected demographic surveys specifying ethnic group composition of Central and West African cities, at several points in time. We generated lower and upper estimates of circumcision frequency in urban male adults of each ethnic group, based on the method explained in Text S2 and implemented in Dataset S1. We then computed the distribution of adults in cities by circumcision classes displayed in Figure 3, and the estimates of circumcision frequencies displayed in Figure 4. The full tables of ethnic composition, and the supporting references of this study are in Text S2 and Dataset S1.

### Simulation study

We developed a stochastic, individual-based simulation model to track the initial spread of HIV over a dynamic network of sexual contacts. The model distinguished married and single men and women, “*femmes libres*” and commercial sex workers (CSW). During the timeframe of the simulations (one year), the population was assumed to be constant (no birth, death or migration). The sexual network consisted of stable (spousal) links, short-term links and male visits to CSWs. Stable links were fixed throughout a simulation; short-term links were allowed to form and break up at each time step, and involved both married and single men and women, and *femmes libres*. Network parameters for married and single men and women were set according to modern studies from Yaoundé (the closest available survey location) (see Table 4 and references therein). *Femmes libres* were assigned with replacement to the remaining “open” short links of men: their sexual promiscuity was thus governed by the sex ratio (the shortage of short links by single and married women compared with short links of men) in each scenario. The number of CSWs was also automatically set in each scenario to match the demand for CSW visits.

Simulations had a time step of one week. All runs were preceded by an initialization phase restricted to link formation and breakup until the sexual network settled to a steady state. The initial HIV infection was then introduced into a single male to reflect the most likely source of a bushmeat hunter, and the spread of the virus was followed for a year. At each time step, sexual contacts were generated randomly over stable and short links; CSW visits involved a single sexual contact. HIV transmission was restricted to acute infection; its probability per contact was modified by GUD and circumcision status. Transmission rates were scaled according to estimates on modern HIV (Table 4), assuming multiplicative modifier effects and bounded from above by a maximum allowed rate. The basic acute transmission rate of early HIV was per default assumed to be lower than that of modern HIV, but higher than modern rates of chronic transmission. Circumcision status in men was fixed for the duration of the simulations. GUD dynamics was not modeled explicitly: healed genital ulcers were replaced randomly from the appropriate class of individuals. The age of GUD episodes and HIV infections was updated at the end of each time step. Chronic HIV infection was per default not transmissible, but protected against repeated acute infection. Population figures were set to reflect historical scenarios as described in the main text. Simulations were implemented in the R software package [137]. All code is provided in Text S3.

## Supporting Information

Figure S1 The biogeography of epidemic HIV strains in Central Africa (A), and West Africa (B). The ranges of the primates that were the source of SIVs that gave rise to HIV strains are indicated (based on [2], [11]–[13]). The circles mark the locations where SIVs most closely matching HIV-1 group M [2] and HIV-2 groups A and B [9] were found. Compelling evidence suggests that the countries indicated in red were the most likely epicenters of particular HIV groups [13]–[17], [77], [78]. The references cited in this legend are listed in the main article.
(8.73 MB TIF)

Table S1 Modern levels of male circumcision in relevant countries of Central and West Africa.
(0.02 MB PDF)

Table S2 The coverage of male circumcision information attained by our ethnographic survey.
(0.02 MB PDF)

Table S3 Killing and consumption of apes/monkeys in Central and West Africa.
(0.06 MB PDF)

Table S4 Summary of the simulations outcomes for the four historical scenarios.
(0.04 MB XLS)

Table S5 Summary of simulation outcomes for combinatorial variants of the Kinshasa 1919 scenario.
(0.05 MB XLS)

Text S1 GUD incidences in Leopoldville/Kinshasa.
(0.14 MB PDF)

Text S2 Circumcision prevalences in Central and West Africa.
(0.12 MB PDF)

Text S3 Source code of the simulations.
(0.03 MB ZIP)

Dataset S1 Circumcision classes and frequencies in Central and West African cities.
(0.67 MB XLS)
