# Supplementary figures and images for: High GUD Incidence in the Early 20th Century Created a Particularly Permissive Time Window for the Origin and Initial Spread of Epidemic HIV Strains
Source: PLoS One. 2010 Apr 1;5(4):e9936. doi: 10.1371/journal.pone.0009936 (PMC2848574; doi:10.1371/journal.pone.0009936)

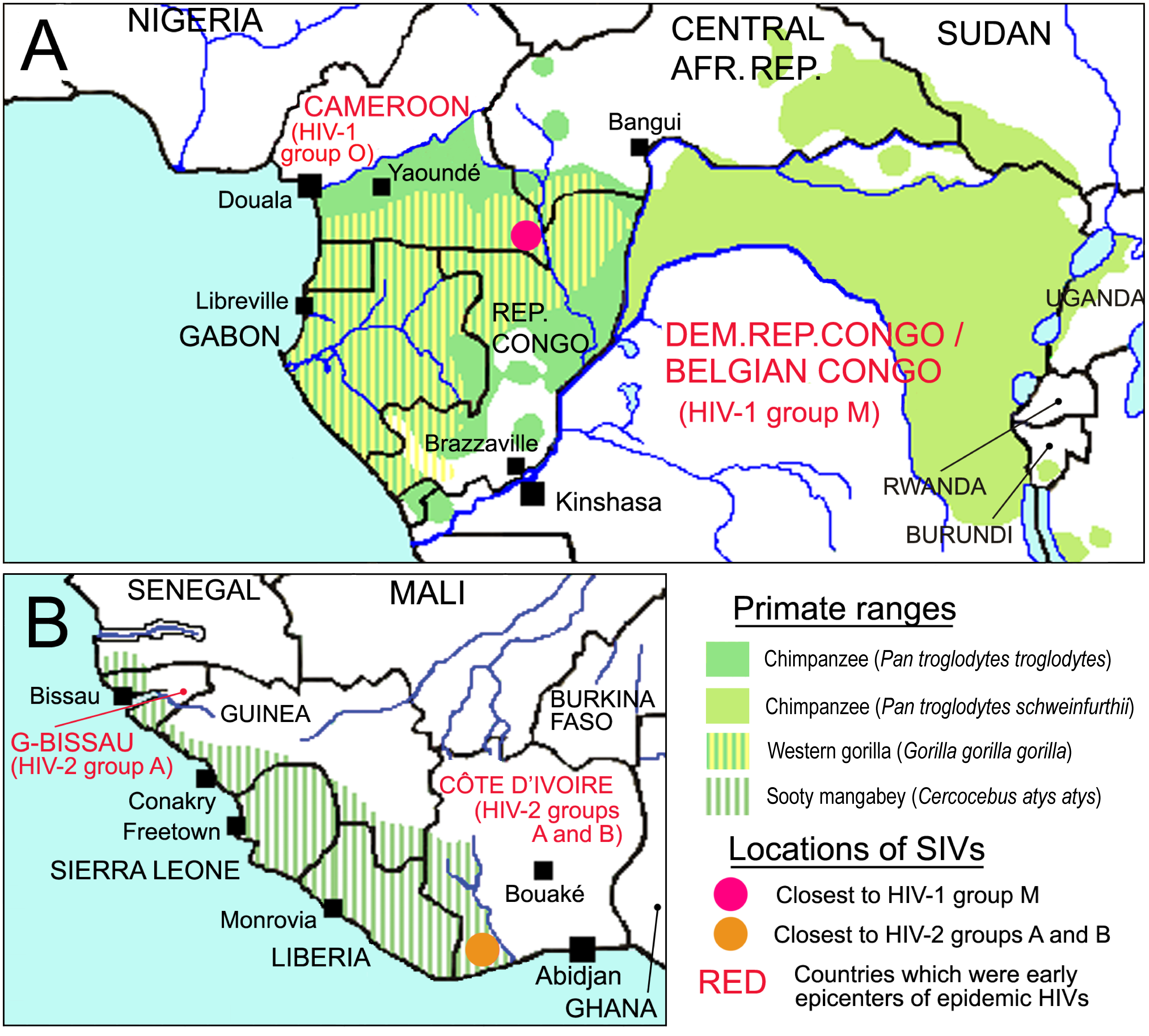

Supplement: Figure S1 — The biogeography of epidemic HIV strains in Central Africa (A), and West Africa (B). The ranges of the primates that were the source of SIVs that gave rise to HIV strains are indicated (based on [2], [11]–[13]). The circles mark the locations where SIVs most closely matching HIV-1 group M [2] and HIV-2 groups A and B [9] were found. Compelling evidence suggests that the countries indicated in red were the most likely epicenters of particular HIV groups [13]–[17], [77], [78]. The references cited in this legend are listed in the main article. (8.73 MB TIF) [file pone.0009936.s001.tif]
