# Supplementary material for: High GUD Incidence in the Early 20th Century Created a Particularly Permissive Time Window for the Origin and Initial Spread of Epidemic HIV Strains
Source: PLoS One. 2010 Apr 1;5(4):e9936. doi: 10.1371/journal.pone.0009936 (PMC2848574; doi:10.1371/journal.pone.0009936)
Supplement: Table S1 — Modern levels of male circumcision in relevant countries of Central and West Africa. (0.02 MB PDF) [file pone.0009936.s002.pdf]

Country	Circumcision frequency	Year of observation	Refs. (see main article)
Cameroon	93.0% a	2004	DHS [89]
DRC	97.4%	2007	DHS
Rep. Congo	98.4%	2005	DHS
CAR	93–98% b		c
Gabon	97–99% b		c
Equat. Guinea	97–99% b		
Rwanda	9.7%	2005	DHS
Burundi	8–12% b		
Uganda	34.7%	2006	DHS
Tanzania	69.4%	2003	DHS
Senegal	98.2%	2005	DHS
Guinea-Bissau	79–97%	2000–2006	[90,91]
Guinea	98.7%	2005	DHS
Sierra Leone	97–99% b		
Liberia	97.7%	2007	DHS
Côte d'Ivoire	96.0%	2006	DHS
Ghana	95.0%	2003	DHS
Mali	97.7%	2006	DHS
Burkina-Faso	89.7%	2003	DHS

Table S1. Modern levels of male circumcision in relevant countries of Central and West Africa.

Most data is based on the Demographic and Health Surveys (DHS) [89]. The references cited in this table are listed in the main article.

a In the south, southwest, east and center regions it is >97% [89]. **b** Estimate based on the cultural similarity between the ethnic groups, and cultural trends in circumcision, of these countries, and those of neighboring countries for which data is known. **c** For these countries, DHS surveys exist, but they did not survey circumcision.
