# Supplementary material for: High GUD Incidence in the Early 20th Century Created a Particularly Permissive Time Window for the Origin and Initial Spread of Epidemic HIV Strains
Source: PLoS One. 2010 Apr 1;5(4):e9936. doi: 10.1371/journal.pone.0009936 (PMC2848574; doi:10.1371/journal.pone.0009936)
Supplement: Table S2 — The coverage of male circumcision information attained by our ethnographic survey. (0.02 MB PDF) [file pone.0009936.s003.pdf]

Country	Number of important ethnic groups [a]	Number of ethnic groups with data on circumcision (Revised Atlas)	Number of ethnic groups with data on circumcision after our study [b]
DRC	93	49	81
Rep.Congo	17	6	16
CAR	26	8	19
Gabon	11	3	11
Equat.Guinea	7	3	6
Cameroon	70	35	59
Senegal	11	8	11
Guinea-Bissau	11	4	10
Guinea	14	10	13
Sierra Leone	9	4	9
Liberia	16	7	15
Côte d' Ivoire	33	15	28
Total	318	152	278

Table S2. The coverage of male circumcision information attained by our ethnographic survey.

Our ethnographic survey greatly increased coverage of male circumcision information per ethnic group (last column) in relation to the Revised Ethnographic Atlas (Gray 1999) [93]. The references cited in this table are listed in the main article.

[a]The count includes all groups with more than 20,000 people (based on Gordon (2005) [94]), and additional ones for which we gathered information; no group is counted in more than one country in the table. [b]That is, including both the statements about circumcision present in the Revised Ethnographic Atlas (Gray 1999) [93] and those we collected in our survey of additional ethnographic reviews, and primary articles/books (which references are listed in Text S2).
