# Supplementary material for: High GUD Incidence in the Early 20th Century Created a Particularly Permissive Time Window for the Origin and Initial Spread of Epidemic HIV Strains
Source: PLoS One. 2010 Apr 1;5(4):e9936. doi: 10.1371/journal.pone.0009936 (PMC2848574; doi:10.1371/journal.pone.0009936)
Supplement: Table S3 — Killing and consumption of apes/monkeys in Central and West Africa. (0.06 MB PDF) [file pone.0009936.s004.pdf]

Ethnic groups	Region and country	Killing	Consu mption	Time	Refs
**Part I**	**Hunting of chimpanzees and gorillas in Central Africa**				
Ngbandi	Ubangi region, north Democratic Republic of Congo (DRC)	Yes	Yes	1920–55	14
Ngbandi	Ubangi region, north DRC	Yes	Yes	1970	**a**
Ngbaka	Northwest DRC	Yes	Yes	1920–55	14
Ngombe	Équateur province, DRC	Yes	Yes	1950	119, **b**
Mongo	Équateur province, DRC	Yes	Yes		**c**
Ngando	Wamba region, central DRC	Seldom	No	1900–80	112
Topoke, Lokele, Mbole, Boyela, Bosaka, Mbesa	Eastern part of Équateur province, DRC	Yes	Yes		112
Tutsi, Hutu	Rwanda, Burundi		No	1910	16
Tutsi, Hutu	Rwanda, Burundi		No	2000	111
Tutsi, Hutu	Burundi	Seldom	No	1920	113
Twa Pygmies	Virunga mountains, Rwanda	Seldom	No	1967–82	115
Shi-Havu	Bukavu area, east DRC	Seldom	No	1920	114
Tembo	North Kivu region, east DRC	Yes	Probable		120
Lega-Mwenga	Mwenga area, east DRC	Yes	Probable		120
Mberé	Ewo region, Congo	Probable		1955	121
Bomitaba	Northeast Congo	Yes	Yes	1920	122
Aka Pygmies	North Congo, SW Central African Republic (CAR)	Yes	Seldom	1995	108
Bongili, Kaka, Pomo, Bomali	North Congo	Yes	Yes	1995	108
Bomitaba, Bondongo, Kaka, Aka, Bondjo	Motaba and Likouala aux Herbes regions, north Congo	Yes, intensely	Yes, favoured	1992	123, 124
Mbochi, Mboko	Cuvette region, Congo	Yes	Probable		120
Bomali, Pomo, Kaka	Ouesso area, north Congo	Seldom	Seldom	1900	118
Bakwele	Northwest Congo	Yes, intensely	Yes, favoured	1940–80	120, 125
Bakwele, Djem, Fang	Northwest Congo	Yes	Yes	2000	110
Bakota	Odzala region, Congo	Yes	Yes		120
Yombe, Lumbu	West Congo and SW Gabon	Yes	Yes		120
Gbaya	Kadei area, west CAR	Yes	Yes	1910	16
Mbimou	Nola region, CAR, Yokadouma region, Cameroon	Yes, intensely	Yes, favoured	1905	126
Fang	Northwest Gabon	Yes	Yes	1860–90	116, 127
Fang	Moyen Ogooué, Gabon	Yes	Yes	1930	128
Fang, Ntumu	Woleu-Ntem region, north Gabon and south Cameroon	Yes	Yes		129
Pahouin group	Unspecified location	No	No	1950	130
Mpongwé	Estuaire, litoral Gabon	No	No	1860	116, 117
Orungu	Lower Ogooué, Gabon	Yes	Probable		131
Nkomi, Bakalai	Lower Ogooué, Gabon	Yes	Yes	1860	117
Shekiani, Fang	Equatorial Guinea	Yes	Yes	1860	117
Beti, Yaunde	Central Cameroon	Yes	Probable	1965	40
Beti, Yaunde, Mvele	Central Cameroon	Yes	Yes	1920–40	129
Bulu	South Cameroon	Yes	Probable	1890	127
Bulu	South Cameroon	Yes	Yes		120, **d**
Bassa	Nyong-Kelle region, Cameroon	Yes	Yes		**d**
Dzimou, Badjoué	Dja forest and south central Cameroon	Yes	Yes	1920	132

Bomali, Dzimou, Essel, Boman, Konabembé	Moloundou area, SE Cameroon and NW Congo	Yes	Yes	1910	106
Medjimé	Southeast Cameroon	Yes, intensely	Yes, favoured	1920–40	129
Baka Pygmies	Southeast Cameroon	Yes		1940	107
Baka Pygmies	Southeast Cameroon	Yes	Seldom	2000	109, 110
Banen	Bafia region, Cameroon	Seldom	Seldom	1950	133
Banyang	West Cameroon	Limited	Limited	1940–60	120
Chamba	Faro division, north Cameroon	Limited	Limited		120
Bamileke	Grassfields region, NW Cameroon	No	No		**d**
**Part II**	**Hunting of monkeys in West Africa**				
Badyara	Northeast Guinea-Bissau, and nearby Guinea and Senegal	Yes	Yes	1910	134
Manjaco	Northwest Guinea-Bissau		No	1955	56
Mancanha	Northwest Guinea-Bissau	Yes	Yes	1940	135
Bijagó	Bijagó islands, Guinea-Bissau	Yes	Yes	1930	30
Coniagui, Bassari	Koundara region, Guinea, and southeast Senegal	Yes	Yes	1910	134
Baga	Coastal Guinea	Yes	Yes	1900	136
Kpelle	Central Liberia	Yes	Yes	1925	26, 137
Sapo	East Liberia	Yes	Yes	1928	27
Loma	Loffa region, Liberia	Yes	Yes	1928	27
Mano, Gio-Dan	Nimba region, Liberia	Yes	Yes	1928	27
Beté	Daloa, Gagnoa, and Guiberoa regions, Côte d'Ivoire	Yes	Yes	1940–60	22, 138
Beté	Daloa region, Côte d'Ivoire	Yes, intensely	Yes	1925	139
Niaboua	West central Côte d'Ivoire	Yes	Yes	1940	138
Krumen	Southwest coast, Côte d'Ivoire	Yes	Yes	1940	140
Agni	Southeast coast, Côte d'Ivoire	Yes, intensely	Yes, favoured	1890	25

Table S3. Killing and consumption of apes/monkeys in Central and West Africa.

A compilation of ethnographic and other literary references on the hunting of/feeding on: 1) chimpanzees and/or gorillas in Central Africa (Part I); any monkeys in West Africa (Part II). For many ethnic groups/populations, killing of the relevant animals was practiced without eating their meat (e.g., the Aka Pygmies killed apes to supply bushmeat to others; the Tutsi and Hutu did it seldom only to protect crops). For additional information, see Text S2. The references cited in this table are listed in Text S2.

a Personal communication from Christiaan Van Goethem (experienced doctor who worked in the DRC). **b** Personal communication from Alvin Wolfe (Northwestern Univ). **c** Personal communication from Honoré Vinck (Aequatoria, Belgium and DRC). **d** Unpublished report supplied by Flavien Ndonko (Gesellschaft für Technische Zusammenarbeit, Yaoundé, Cameroon).
