# Supplementary material for: High GUD Incidence in the Early 20th Century Created a Particularly Permissive Time Window for the Origin and Initial Spread of Epidemic HIV Strains
Source: PLoS One. 2010 Apr 1;5(4):e9936. doi: 10.1371/journal.pone.0009936 (PMC2848574; doi:10.1371/journal.pone.0009936)
Supplement: Text S1 — GUD incidences in Leopoldville/Kinshasa. (0.14 MB PDF) [file pone.0009936.s007.pdf]

High GUD incidence in the early 20th century created a particularly permissive time window for the origin and initial spread of epidemic HIV strains

João Dinis de Sousa[1*], Viktor Müller[2], Philippe Lemey[1], Anne-Mieke Vandamme[1,3]

1 Laboratory for Clinical and Evolutionary Virology, Rega Institute for Medical Research, Katholieke Universiteit Leuven, Leuven, Belgium

2 Institute of Biology, Eötvös Loránd University, Budapest, Hungary

3 Centro de Malária e Outras Doenças Tropicais, Instituto de Higiene e Medicina Tropical, Universidade Nova de Lisboa, Lisboa, Portugal

* Corresponding author. E-mail: joao.sousa@rega.kuleuven.be

Supplementary Text S1: GUD incidences in Leopoldville/Kinshasa

This supplementary text presents the information on: 1) GUD incidences in Leopoldville/Kinshasa in the period 1919–58, used for Figure 1 in the main text, and 2) additional information about GUDs and STDs in the same city before 1919. The reference numbering used in this supplementary text corresponds to its own reference list.

1. GUD incidences in Leopoldville/Kinshasa in the period 1919–58

We surveyed all reports on the period 1919–58 in the Afrika Archief (Federale Overheidsdients – Buitenlandse Zaken, Buitenlandse Handel en Ontwikkelingssamenwerking (Ministry of Foreign Affairs), Brussels) of the categories that are about health and report disease cases in Leopoldville. Table TS1-1 lists the archival sources consulted.

Data coverage was very inconsistent between the years, with the reports often changing names, and changing the way they discriminated regions/districts. For example, some of the health reports at the level of the colony have disease tables that stratify by province only, others stratify by district within each province, and many only present the numbers for the colony as a whole. We were interested in Leopoldville, which formed a district on its own: District Urbain de Leopoldville (DUL). Numbers of disease cases in the DUL appeared in reports at the DUL level itself, and sometimes in reports at the province level (Congo-Kasai province), or at the colony level.

Based on the disease case data, we built Tables TS1-2 for syphilis, TS1-3 for chancroid, and TS1-4 for *lymphogranulomma venereum* (LGV). They serve as basis for Figure 1 of the main text. The lacunae in tables stem from many reasons. For some years, we could not find relevant reports in the Afrika Archief after months of research. For many health reports, or health sections of reports, qualitative statements appeared instead of numbers of disease cases. In some cases, these numbers appeared for many diseases, but not for those we were interested in. There was a clear bias in favor of syphilis, compared to chancroid and LGV, the latter diseases being simply omitted in many of the tables which listed STDs including syphilis. We chose to start our chart in the year 1919, because the reports were to a large extent incomplete in the previous years.

Detection and treatment of STDs in the city was done by three different “health systems”: the Public Sector (including public hospitals, dispensaries, and the action of doctors working for the Public Sector), the Industrial Hygiene (health care provided by private firms), and a Red Cross anti-venereal dispensary (Croix Rouge du Congo à Leopoldville), which opened in 1929. For each year, we sum up the cases of the three systems, to calculate incidences in adults. For some years, we made estimates for one health system, which lacked reported data, but we used this procedure with parsimony.

The proportion of primo-secondary syphilis (PSS) cases was reported in many documents surveyed, and showed a marked temporal decline from 43–63% in the period 1925–33 to 1–9% in the period 1949–58, in line with theoretical studies of syphilis epidemics [1].

The Industrial Hygiene record is very incomplete, but our estimates in some years are similar to the values obtained for nearby years (Table TS1-2). The low numbers of syphilis cases in the Public Sector in the 1950s results from the fact that, at these times, most STD cases detected in this system were forwarded to the Croix Rouge dispensary [2–4].

In conclusion, the decline of two orders of magnitude in PSS incidences between the periods 1919–35 and 1953–58 is a robust finding. The same result is obtained if we only consider the Croix Rouge cases, the most complete record that includes the majority of cases of the city, and for which we did not have to include any estimates in the table (Table TS1-2).

Inven-tory	Box / Folder	Types of document consulted	Years
A39	RA/MED 1 to 5	Official annual health reports, colony level	1925–1958
A39	RA/MED 6	Croix Rouge du Congo, annual reports (includes the venereal disease clinic in Leopoldville)	1936–53
A39	80	Miscellaneous documents and reports (colony level and Leopoldville/Stanley Pool level)	1910–12
A39	81	Miscellaneous documents and reports (colony level and Leopoldville/Stanley Pool level)	1913–25
A39	82	Miscellaneous documents and reports (colony level, provincial level, and District Urbain de Leopoldville (DUL) level)	1926–28
A39	87	Official health reports (Congo-Kasai province level, some data at Leopoldville/DUL level)	1926–28
A39	88	Official health reports (Congo-Kasai province level, some data at Leopoldville/DUL level)	1929–31
A39	89	Official health reports (Congo-Kasai province level, some data at Leopoldville/DUL level)	1932–33
A39	135	Official reports, including health (Leopoldville province level, some data at DUL level)	1935–38, 1940
A39	252	Official reports, including health (Leopoldville province level, some data at DUL level)	1939, 1945–49
A39	253	Official reports, including health (Leopoldville province level, some data at DUL level)	1950–52
A39	254	Official reports, including health (Leopoldville province level, some data at DUL level)	1953–58
A39	RA/MED 17	Official health reports (Leopoldville province level, some data at DUL level)	1952–54
A39	RA/MED 17	Official health reports (Leopoldville province level, some data at DUL level)	1956–58
A39	RA/MED 46	Official health reports (DUL level)	1931–51
A11	841	Miscellaneous documents and reports (Leopoldville/Stanley Pool level)	1904–11
GG	5398	Croix Rouge du Congo, annual reports (includes the venereal disease clinic in Leopoldville)	1930–35
GG	16829	Miscellaneous documents and reports (Leopoldville/Stanley Pool level)	1910
GG	16807	Miscellaneous documents and reports (Leopoldville/Stanley Pool level)	1911

Table TS1-1. Archival sources consulted to obtain the GUD data for Leopoldville/Kinshasa.

These archival sources were consulted in the Afrika Archief (Federale Overheidsdients – Buitenlandse Zaken, Buitenlandse Handel en Ontwikkelingssamenwerking (FO-BZBHO) (Ministry of Foreign Affairs, Brussels)) to obtain the data presented in Tables TS1-2, TS1-3, and TS1-4 of this supplementary information, and thus of Figure 4 of the main text.

			Syphilis cases in the city				Incid. in adults (%)	
Year	Adult Popul.	% of PSS	Croix Rouge	Public Sector	Industrial Hygiene	Total used in chart	Syphilis Incidence	PSS Incidence
1919	11974	75[a]	0	386	150–180[d]	536–566	4.48–4.73	3.36–3.55
1920	17420		0					
1921			0					
1922	18133		0					
1923	18000	65[c]	0	490	250–350[d]	740–840	4.11–4.67	2.67–3.03
1924	19500		0					
1925	21752	42.7	0	907	270–370[d]	1177–1277	5.41–5.87	2.31–2.51
1926	27125	55.1	0					
1927	30681	63.2	0	684	400–480[d]	1084–1164	3.53–3.79	2.23–2.40
1928	37841	60[c]	0	1016	460–560[d]	1476–1576	3.90–4.16	2.34–2.50
1929	43554	57.6	394	1276	500–600[d]	2170–2270	4.98–5.21	2.87–3.00
1930	32594	51.6	29	798	450–560[d]	1277–1377	3.92–4.22	2.02–2.18
1931	29205	57.7	923	797	436	2156	7.38	4.26
1932	25087	54.8	294	765	254	1313	5.23	2.87
1933	18596	51.9	879	775	200–310[c]	1854–1964	9.97–10.6	5.18–5.49
1934	21152		650					
1935	21518	44[c]	616	661	200–310[c]	1477–1587	6.86–7.33	3.02–3.25
1936	23729		338					
1937	27042		365					
1938	31279		562					
1939	29921	30[c]	612	450–560[b]	256	1318–1428	4.40–4.77	1.32–1.43
1940	34976	27[c]	786	650–800[b]	324	1760–1910	5.03–5.46	1.36–1.47
1941	37645				284			
1942	49648		1030					
1943	59223		942					
1944	59174		1000					
1945	70780	10.0	899					
1946	79467	8.5[c]	1263	720–880[c]	477	2460–2620	3.10–3.30	0.263–0.280
1947	85670	6.8[c]	1386	800–870[b]	422	2608–2678	3.04–3.13	0.207–0.213
1948	87116		1095					
1949	103441	3.7	1096	500–600[b]	233	1829–1929	1.77–1.86	0.065–0.068
1950	126845	5.6	1093		157			
1951	144142	7.7	649		146			
1952	139758							
1953	157878	3.5[c]	2142	200–240[b]	80–140[e]	2422–2522	1.53–1.60	0.054–0.056
1954	174360	1.2	863	130–170[b]	75–125[f]	1068–1168	0.60–0.66	0.007–0.008
1955	174697							
1956	188061	2.2	714					
1957	196531	5.5[c]	419	66	75–125[f]	560–610	0.28–0.31	0.016–0.017
1958	205000	9.5	685	157	75–125[f]	917–967	0.45–0.47	0.043–0.045

Table TS1-2. Adult population, and syphilis cases in Leopoldville, in the period 1919–58.

[a] We estimate here 75% of PSS among syphilis cases, intermediate between the values of 43–63% of the period 1925–33, and a direct report of 97% PSS in the syphilis cases of 1912 [5]. [b] Estimate for the city resulting from the application of the average proportion of city cases/province cases (Public Sector only) observed in neighboring years to the available number of cases for the Congo-Kasai province. [c] Interpolation. [d] These estimates were made applying to the city adult male population syphilis incidences only slightly higher than the 1.6–2.1% Industrial Hygiene incidences in males seen in 1931–32, and allowing for the same slight decline throughout the period 1919–29 seen in the other systems. [e] Estimate for the city resulting from the application of the average proportion of city cases/province cases (Industrial Hygiene only) observed in neighboring years to the available number of cases for the Congo-Kasai province. [f] Estimate taking into account the same slight decline between 1953 and 1954–58 seen in the other sectors.

		Chancroid cases in the city				Incidences in adults (%)
Year	Adult Popul.	Croix Rouge[a]	Public Sector	Industrial Hygiene	Total	
1919	11974					
1920	17420					
1921						
1922	18133					
1923	18000					
1924	19500					
1925	21752					
1926	27125					
1927	30681					
1928	37841					
1929	43554					
1930	32594	37	38	140–220[e]	215–295	0.66–0.91
1931	29205	130–200[d]	215	167	512–582	1.75–1.99
1932	25087	100–170[d]	62	117	279–349	1.11–1.39
1933	18596	70–130[d]	41	65–125[e]	176–296	0.95–1.59
1934	21152					
1935	21518	43	102	70–130[e]	215–275	1.0–1.28
1936	23729					
1937	27042	299				
1938	31279	271				
1939	29921	65	225–345[b]	109	399–519	1.33–1.73
1940	34976	47	80–160[b]	70	197–277	0.56–0.79
1941	37645			85		
1942	49648	35				
1943	59223	305				
1944	59174	127				
1945	70780	33	150–280[b]	65–105[e]	248–418	0.35–0.59
1946	79467	39	130–250[c]	81	250–370	0.31–0.47
1947	85670	152	120–220[b]	110	382–482	0.45–0.56
1948	87116					
1949	103441	97	40–90[b]	71	208–258	0.20–0.25
1950	126845	106		39		
1951	144142	50		109		
1952	139758			5		
1953	157878			6		
1954	174360	1				
1955	174697					
1956	188061			7		
1957	196531	21	25	3–10[e]	49–56	0.025–0.029
1958	205000	31	32	3–10[e]	66–73	0.032–0.036

Table TS1-3. Adult population, and chancroid cases in Leopoldville, in the period 1919–58.

[a] Described as “genital ulcerations in men other than those caused by syphilis” [6]; we assume they probably were mostly chancroid, because genital herpes was rare at that time (as can be inferred from [7]), and LGV’s ulcers in men are barely noticed and seldom lead to consultations. [b] Estimate for the city resulting from the application of the average proportion of city cases/province cases (Public Sector only) observed in neighboring years to the available number of cases for the Congo-Kasai province. [c] Interpolation. [d] These estimates were made applying to the city adult male population incidences equal to the average incidences in males seen in 1935–40 (in Croix Rouge only). [e] Estimate for the city resulting from the application of the average proportion of city cases/province cases (Industrial Hygiene only) observed in neighboring years to the available number of cases for the Congo-Kasai province.

		LGV cases in the city			Incidences in adults (%)
Year	Adult Popul.	Croix Rouge[a]	Public Sector	Total	
1919	11974				
1920	17420				
1921					
1922	18133				
1923	18000		152	152	0.84
1924	19500				
1925	21752		208	208	0.96
1926	27125		189	189	0.70
1927	30681				
1928	37841		42	42	0.11
1929	43554		84	84	0.19
1930	32594		95	95	0.29
1931	29205		157	157	0.24
1932	25087		121	121	0.48
1933	18596		96	96	0.52
1934	21152				
1935	21518		138	138	0.64
1936	23729		41	41	0.17
1937	27042				
1938	31279		206	206	0.66
1939	29921				
1940	34976				
1941	37645				
1942	49648				
1943	59223				
1944	59174				
1945	70780	22	50–150[b]	72–172	0.10–0.24
1946	79467	39	50–150[b]	89–189	0.11–0.24
1947	85670				
1948	87116				
1949	103441				
1950	126845				
1951	144142				
1952	139758		18	18	0.013
1953	157878		25	25	0.016
1954	174360		19	19	0.011
1955	174697				
1956	188061		22	22	0.012
1957	196531		1	1	0.00051
1958	205000	5	13	18	0.0088

Table TS1-4. Adult population, and LGV cases in Leopoldville, in the period 1919–58.

[a] The Croix Rouge reports only mention LGV cases in 1945, 1946, and 1958; based on the reported cases of venereal diseases other than syphilis, chancroid, and gonorrhea, they should be no more than a few dozens in most years. The Industrial Hygiene reports don't specify LGV. [b] Interpolation.

2. GUDs and STDs in Leopoldville before 1919

The health reports from before 1919 were very incomplete, often mentioning only syphilis and blennorrhagia, covering few years, and not covering all health care providers. Table TS1-5 presents the STD cases in Africans treated in the Leopoldville hospital.

	Hospitalized STD cases in Africans			
Year	Syphilis	Blennorrhagia	Chancroid	LGV
1906	16	4	4	
1907	26			
1908	36	13		
1909				
1910	34	9		5
1911	27	11		11
1912	16	2		
1913				
1914				
1915	12	2		

Table TS1-5. STD cases in Africans treated in the Leopoldville hospital, in the period 1906–15.

The small numbers that appear reflect the fact that only the most severe cases of STDs were being hospitalized [8]. For each STD, the total number of cases being treated in the city was much higher than these values [5,8–11]. Contrasting with the small numbers of hospitalized blennorrhagia cases (Table TS1-5), an author says that, by 1910, almost all Africans in the city were infected by gonorrhea and suffered from intermittent urethritis as a consequence [9].

Therefore, Table TS1-5 is not comparable to the previous tables covering the period 1919–58, and does not permit the calculatation of meaningful incidences. We therefore limited our analysis of GUD incidences (Figure 1 of the main text) to the period 1919–58.

However, some reports and articles we obtained demonstrate high GUD and other STD incidences in the period 1906–15. Mouchet and Dubois treated 68 syphilis cases in Leopoldville, almost all primo-secondary (PSS), during an unspecified number of months of 1911 and 1912 [5]. Other evidence comes from reports of monthly medical visits to the men working for the public sector in Leopoldville. In the first four months of 1911, the public sector workers (averaging 1,113 men), generated 30 syphilis and 475 blennorrhagia entries in the monthly reports [10,11]. Assuming no overlap of syphilis cases in consecutive months (which is reasonable, since a review of the treatments made in that year showed that PSS symptoms were cleared in no more than two weeks [5]), these men had 30 independent PSS cases in four months, which implies an annualized incidence of 8.1%. This incidence is three times higher than the incidences we obtained for the period 1919–29 (Table TS1-2). In addition, at any given moment of observation, 96–146 of these men (9.1–12.5% of the work force in each month) had active blennorrhagia [10,11]. These public sector workers numbered 1,050–

1,300 by this time [9–11], and so they were a high proportion of the 3,000–4,000 adults then living in Leopoldville [12]. We conclude that GUD and other STD incidences in Leopoldville in the years around 1911 were probably higher than in the period 1919–29.

References

1. Garnett GP, Aral SO, Hoyle DV, Cates W, Anderson RM (1997) The natural history of syphilis: implications for the transmission dynamics and control of infection. Sex Transm Dis 24: 185–200.

2. Services Médicaux (1952) Rapport annuel. Leopoldville, Belgian Congo: Services Médicaux, Province de Leopoldville. Afrika Archief (FO-BZBHO, Brussels), Inventory A39, Box RA/MED 17.

3. Service d'Hygiene Publique (1958) Rapport annuel, ville de Leopoldville. Leopoldville, Belgian Congo: Service d'Hygiene Publique, Province de Leopoldville. Afrika Archief (FO-BZBHO, Brussels), Inventory A39, Box RA/MED 18.

4. Bruaux P, Cerf J, Lebrun A (1957) La lutte contre les affections vénériennes à Léopoldville. Ann Soc Belg Méd Trop 37: 801–813.

5. Mouchet R, Dubois A (1913) Le traitement du Pian et de la Syphilis par le Salvarsan dans la pratique indigène. Bull Soc Pathol Exot Fil 6: 14–19.

6. Croix Rouge du Congo (1928–54) Rapport annuel [1928 through 1954]. Brussels: Croix Rouge de Belgique.

7. O'Farrell N (1999) Increasing prevalence of genital herpes in developing countries: implications for heterosexual HIV transmission and STI control programmes. Sex Transm Infect 75: 377–84.

8. Houssiau D (1910) Rapport sur le fonctionnement de l'hôpital des noirs de Léopoldville pendant l'année 1910. Leopoldville, Belgian Congo. Afrika Archief (FO-BZBHO, Brussels), Inventory A39, Box 80.

9. Service Médical (1910) Note explicative du relevé statistique des visites médicales mensuelles (Novembre-Décembre 1910). Leopoldville, Belgian Congo. Afrika Archief (FO-BZBHO, Brussels), Series GG, Folder GG 16829.

10. Service Médical (1911) Résultats des examens médicaux mensuels pour les mois de Janvier et Février 1911. Leopoldville,

Belgian Congo. Afrika Archief (FO-BZBHO, Brussels), Inventory A11, Box 841.

11. Service Médical (1911) Note explicative du relevé statistique des visites médicales mensuelles (Mars–Avril-Mai 1911). Leopoldville, Belgian Congo. Afrika Archief (FO-BZBHO, Brussels), Inventory A11, Box 841.

12. Mouchet R (1913) La tuberculose à Léopoldville (Congo Belge). Bull Soc Pathol Exot Fil 6: 55–68.
