# Supplementary material for: High GUD Incidence in the Early 20th Century Created a Particularly Permissive Time Window for the Origin and Initial Spread of Epidemic HIV Strains
Source: PLoS One. 2010 Apr 1;5(4):e9936. doi: 10.1371/journal.pone.0009936 (PMC2848574; doi:10.1371/journal.pone.0009936)
Supplement: Text S2 — Circumcision prevalences in Central and West Africa. (0.12 MB PDF) [file pone.0009936.s008.pdf]

High GUD incidence in the early 20th century created a particularly permissive time window for the origin and initial spread of epidemic HIV strains

João Dinis de Sousa[1*], Viktor Müller[2], Philippe Lemey[1], Anne-Mieke Vandamme[1,3]

1 Laboratory for Clinical and Evolutionary Virology, Rega Institute for Medical Research, Katholieke Universiteit Leuven, Leuven, Belgium

2 Institute of Biology, Eötvös Loránd University, Budapest, Hungary

3 Centro de Malária e Outras Doenças Tropicais, Instituto de Higiene e Medicina Tropical, Universidade Nova de Lisboa, Lisboa, Portugal

* Corresponding author. E-mail: joao.sousa@rega.kuleuven.be

Supplementary Text S2: Circumcision prevalences in Central and West Africa

This supplementary information presents the data used to construct Figures 2, 3, and 4 (included in the main article), and Supplementary Table S3. It is to be read in conjunction with Dataset S1. In the latter, we present tables of ethnic composition of cities, at several time points. For each ethnic group, and at a given time, we assign a circumcision class, and we estimate circumcision frequency in urban adult males, based on our extensive survey of the ethnographic literature, and thus we compute estimates of circumcision class distribution, and of circumcision frequency for each city/time point.

As described in more detail in Materials and Methods, in the section Circumcision prevalence survey, we collected primary ethnographic articles and books to complement the main ethnographic references [1–3], and modern Demographic Health Surveys (DHS) [4]. Our survey extended the coverage of circumcision information to 87% of the ethnic groups, in relation to the 48% coverage provided by the Ethnographic Atlas [1,2] (Supplementary Table S2). The reference

numbering used in this supplementary text corresponds to its own reference list.

The supplementary information we provide here is divided into the following sections:

1. Temporal trends in circumcision practices and other background information

2. Definition of circumcision classes (relevant to Figure 3 of the main article)

3. Estimation of circumcision frequencies (relevant to Figure 4 of the main article)

4. Ethnically partitioned census tables (relevant to Figures 3 and 4)

5. Additional demographic information

6. Hunting of apes and monkeys (relevant to Suppl. Table S3)

1. Temporal trends in circumcision practices and other background information

Our survey shows that, in both Central and West Africa, the majority of ethnic groups practiced circumcision before colonialism (see Dataset S1). However, the groups not practicing it were numerous. In Central Africa at around 1880, they consisted of: 1) Most groups of northern Cameroon [2,5–7]; 2) Groups living in the Cameroonian area centered around Bafia [7,8]; 3) Almost all groups belonging to the Adamawa-Ubangi language family (this includes almost all groups of the Oubangui-Chari/Central African Republic (CAR) and many groups from the northern parts of Belgian Congo/Democratic Republic of Congo (DRC)) [6,7,9,10]; 4) Most groups of the Orientale province of DRC [7,11–13]; 5) Many groups of the Équateur province of DRC [6,7,14,15]; 6) Most groups of the extreme south of DRC; 7) Almost all groups in Rwanda and Burundi, and nearby DRC areas [2,5,7,11]. In addition, many coastal groups had less common circumcision because of long standing European Christian influence [7,9,16–20]. The high proportion of men coming from the Équateur and Orientale provinces of DRC in Kinshasa in early 20th century (see tables DRC-2, DRC-4, and DRC-5 in Dataset S1) explains the lower circumcision levels in this city compared to Brazzaville and Libreville (Figures 3 and 4 of the main article; Dataset S1).

In West Africa at around 1880, groups not practicing circumcision consisted of: 1) Most groups from the Kru language family in Côte d’Ivoire [7,21,22]; 2) All groups from the Akan language family, and most groups from the northeast of the same country [2,5,6,23–25]; 3) The Kru and Grebo of southwest Liberia [26–28]. In addition, in Guinea-Bissau, several important northern groups performed circumcision in adulthood only, even up to 40 years old [29–32], which generally lowered the circumcision frequency of adults. These patterns explain the singularly low circumcision rates of Ivorian cities (Abidjan and Bouaké) and Bissau in relation to the other West African cities in early 20th century (Figures 3 and 4 of the main article; Dataset S1).

The profound and rapid ethnic mixing promoted by colonialism produced a powerful trend of imitation, with circumcision being adopted by groups previously not practicing it, and generalizing in groups where it was not generalized previously. These changes concentrated in the period 1880–1920 [6,8,9,11–14,33,34]. The end result was nearly universal circumcision in all countries which cities we included in our study (Dataset S1; Figures 3 and 4 of the main article; Supplementary Table S1) [4,35,36]. In countries where

circumcision was rare (e.g., Rwanda, Burundi, and Zambia) it remained so [2,4,5,7].

Christianity produced a decline in circumcision practice only before the start of organized colonialism (circa 1885), and for groups with longstanding contact with Europeans. Because of this, many coastal groups had abandoned it partially or totally by that time [7,9,16–19], and the slave-descended groups that emerged in islands, such as Cabo Verde [37], and São Tomé e Príncipe [38], and in the Caribbean and the Americas [37], abandoned it up to the present day. However, when colonialism started in the continent, Christian missionaries did not discourage circumcision ([39]; personal communication from Tamara Giles-Vernick (Univ of Minnesota and Institut Pasteur, France)). They were more interested in suppressing the initiations and rituals that, for many peoples, accompanied circumcision at puberty [39–42]. These initiations were also complex and costly, and became less affordable under colonial rule. This, associated with the safety of the circumcision operation in health facilities, produced the gradual abandonment of the initiations, and the moving of the operation to immediately after birth or early childhood [4,6,40–45]. This suggests that convergence to the national norm, and moving the operation to childhood, were the main trends at work in 20th century.

Rebuffat (1927) writes that, when circumcision was performed in adolescence or young adulthood, it often led to scars, which increased susceptibility to syphilis [46]. Modern surveys of ritual circumcision in adolescence and young adulthood confirm that keloid scarring, laceration, swelling, and other complications are common [47]. We do not know to which extent these effects could increase susceptibility to HIV, so we did not attempt to model these effects in our simulations.

In a minority of ethnographic sources, the physical procedures of circumcision are described and, in some cases, the description could suggest that the foreskin was only partially removed; this was observed also in recent surveys of ritual circumcision [47]. However, the descriptions are far from conclusive, are made only for a few ethnic groups, and we could never ascertain how a partial circumcision would affect susceptibility to HIV, so we did not attempt to model this effect in our simulations.

2. Definition of circumcision classes

We classified the male populations of the studied cities in classes according to their likelihood of having uncircumcised adult males. For

groups known as practicing it generally, or not practicing it, at a given moment (e.g., a time point in Figures 3 and 4 of the main article) the definition of classes is straightforward. However, for groups known to have adopted circumcision before a given time point, it is unclear what proportion of men were already circumcised at that time point since, after initial adoptions by some subgroups/villages, many years or decades elapsed until full adoption by the whole ethnic group. In these cases, the time elapsed between the first adoptions and a given moment (e.g., a time point in Figures 3 and 4) can be used to define classes reflecting circumcision probability.

Therefore we were interested in the reported times of the first adoptions by ethnic groups. For many groups which adopted circumcision, the ethnographers provide an approximate date of adoption [6,9,11,12,48]. For many additional groups, the ethnographers state, and describe evidence, that adoption had been recent prior to their visit to the tribe studied [8,9,11–13,23,33,34,49–52]. Several authors refer to the temporal order of adoptions of contiguous ethnic groups (or subgroups within a main ethnic group), and describe with some detail “waves” of adoption of circumcision spreading across regions [8,9,11–14,48–50,53–55]. Based on this information, we estimated the dates of adoption of circumcision by groups displayed in Dataset S1.

We assigned to each ethnic group, at a given time, a “circumcision class” that is entirely based on the information provided by the ethnographic sources, and is not dependent on our estimates of circumcision frequencies. The classes are used in Figure 3 of the main article; the related calculations are implemented in Dataset S1. Table TS2-1 shows their definition.

Cl-ass	Colorcode in Fig. 3	Explanation
1	Dark blue	Circumcision is either generalized up to puberty, or its adoption/generalization/move to childhood by the group started more than 45 years before the time of the data point
2	Blue	Circumcision was, less than 45 years before the time of the data point, done in late adolescence/young adulthood by many men
3	Green	Adoption/generalization of circumcision by the group started between 30 and 45 years before the time of the data point
4	Magenta	Adoption/generalization of circumcision by the group started between 15 and 30 years before the time of the data point
5	Orange	Adoption/generalization of circumcision by the group started less than 15 years before the time of the data point
6	Red	Circumcision is absent in the group at the time of the data point
7	Gray	Other situations (e.g., the country of origin and not the ethnic group, is provided, and circumcision is not general in that country)

Table TS2-1. The circumcision classes used in Figure 3 of the main article.

3. Estimation of circumcision frequencies

To construct Figure 4 of the main article, we generated lower and upper estimates of circumcision frequency in urban male adults (age > 20) of each ethnic group, as follows:

i) 0–5% if not practiced.

ii) 97–99% if circumcision is culturally mandatory and performed at or before puberty. This high frequency is supported by the following data: 1) for the groups we put in this category (see Dataset S1) ethnographic sources state that circumcision, even when no initiation was involved, was often necessary for engagement in full adult life, including marriage; the uncircumcised would be mocked, and rejected by women; 2) in the DHS surveys [4] of the countries we studied where ethnicity of the cohorts was recorded, the circumcision frequencies in adults were all above 97.9% (Supplementary Table S1) [4]; 3) in our ethnographic survey, we found two studies which measured circumcision frequency in early or mid 20th century, and they were 100% in adults, in a cohort of Mandjia from Fort Crampel (CAR) in 1900 [10], and 99.6% in adults, in a cohort of Manjaco from Guinea-Bissau in 1960 [56].

iii) 75–85% if commonly performed in late adolescence/early adulthood (in this case, many young men would not have undergone circumcision by the time they were recruited to the cities and other labor settings [40,46,57,58]).

iv) for groups which adopted circumcision in late 19th century or afterwards, and the literature provides an approximate timing of the adoption, we estimated it at 0–20% at that time, and raising linearly to attain 97–99% forty five years later. This is broadly supported for several ethnic groups, for which we could track quantitatively the increase of circumcision prevalence after adoption. For example, the Kru of Liberia were said to be invariably non-circumcised by 1929 [28], and the DHS for Liberia reveals that 98.3% of Liberians born between 1967 and 1976 were already circumcised [4], which implies a very high proportion for the Kru at that time. The Ngbandi from northern DRC adopted circumcision around 1915 [6], and by the early 1970s they were universally circumcised (personal communication from Christiaan Van Goethem (experienced doctor who worked in the DRC)). The Ebrié and other Lagunaire peoples of Côte d’Ivoire were mostly non-circumcised in the 1960s (personal communication from Marc Augé (École d’Hautes Études en Sciences Sociales, France)), and their circumcision levels rose to 96% in 2006 [4]. The examples above set up upper limits for the time elapsed from adoption and universality of about 4–6 decades. The Zande of Faradje, in northeast DRC, were adopting circumcision in 1907 [11], and by 1921 many Zande subgroups had not adopted it yet [7,13]. Several authors reporting on the period between 1920 and 1949 (reviewed in [14]) state that many Ngbandi subgroups were not yet circumcised, and the Ngbandi started to practice circumcision in 1915 [6]. These examples set up lower limits of 2–3 decades for the time between adoption and universality. Therefore we choose as estimate 45 years. We note that the wide initial range of our estimates of circumcision at the adoption time (0–20%) partly incorporates the uncertainty that often exists over this time, and the rate at which it became generalized.

v) if the literature states unequivocally that circumcision is practiced but is far from general, or if there are unresolved conflicting statements by several authors, we estimated it as 40–60% up to the time of this observation, and raising linearly after that, to attain 97–99% thirty years later; in such groups, circumcision was already culturally favored, and the simplicity of the operation performed in childhood in health clinics promoted its universality, as modern DHS surveys [4] demonstrate; also, the cultural pressure to imitate circumcision practice of other groups intensified in the period 1880–1920 [6,7,9,11–14,23,49,53–55,59]).

vi) if the literature states that circumcision was practiced but the influence of Europeans and Christianity in the group had started many generations before the 20th century, we estimated it as 65–85% up to about 1920; many sources give examples of groups in this situation that have abandoned circumcision at least partly [9,16–20,37,38]; after 1920 it raises linearly to attain 97–99% thirty years later; in such groups, circumcision was already culturally favored, and the simplicity of the operation performed in childhood in health clinics promoted its universality, as modern DHS surveys [4] demonstrate; also, the cultural pressure to imitate circumcision practice of other groups intensified in the period 1880–1920 [6,7,9,11–14,23,49,53–55,59]).

Where we lacked information on either the circumcision status of a group, the age at which it is performed, or its generality, we assumed the commonest pattern observed in the neighboring groups.

We implemented this algorithm in Dataset S1. The parameters described in points i)–v) above can be changed in its first sheet, and the spreadsheet recalculates all estimates, updating Figures 3 and 4 automatically.

4. Ethnically partitioned census tables

Table TS2-2 below lists the ethnically partitioned censuses of the studied cities and supporting references. Each line of this table corresponds to a full table included in the companion file Dataset S1, by their order of appearance in the latter. Each table produces both a distribution of men by circumcision classes (used in Figure 3 of the main article), and a circumcision rate estimate (used in Figure 4 of the main article).

For Kinshasa only, the tables we collected more often mentioned the district of origin (within the Belgian Congo) of the inhabitants, rather than their ethnicity. Then, we determined the ethnic composition of each district, by juxtaposing ethnic maps [14,60,61] to an administrative map of the relevant period [62]; the demographic weights of ethnic groups in districts were considered proportional to their respective areas (see Table DRC-2 inside Dataset S1).

City	Year	Sources on ethnic / regional origin of inhabitants	Notes
**Kinshasa** (D.R. Congo)	1958	Spitaels (1959), p.6 [63]	
	1927	AIMO (1927) [64]	
	1919	AIMO (1927) [64]	a)
	1912	Douchet & Dubois (1911–12) [65]	

**Brazzaville** (Rep. Congo)	1950	Balandier (1955), p.37 [66]	
	1932	Subdiv Autonome de Brazzaville (1932) [67]	
	1919	Subdiv Autonome de Brazzaville (1932) [67]	a)
**Bangui** (C.A.Rep.)	1960	Soret (1961), p.68 [68]	
	1949	Lebeuf (1954), p.25 [33]	
	1931	Lebeuf (1954), p.25 [33]	a)
**Libreville** (Gabon)	1953	Lasserre (1958), p.206 [69]	
	1934	Lasserre (1958), p.206 [69]	a)
**Douala** (Cameroon)	1965	Mainet (1985), p.81 [70]	
	1935	Service de Santé (1935) [71]	
	1925	Gouellain (1975), p.286 [72]; Mainet (1985), p.61 [70]; Service de Santé (1935) [71]	b)
**Yaoundé** (Cameroon)	1957	Roubaud (1994), p.9 [73]	
**Bissau** (Guinea-Bissau)	1950	Carreira (1962), p.282 [74]; Junta de Investigação do Ultramar (1950) [75]	
	1940	Província da Guiné (1940) [76]	b)
**Conakry** (Guinea)	1950	Goerg (1990), p.96 [77]	
	1936	Goerg (2006), p.11 [78]; Goerg (1990), p.90 [77]	
	1921	Goerg (1990) [77]; Goerg (2006) [78]	b)
**Freetown** (Sierra Leone)	1963	Harvey (1971), p.98 [79]	
	1953	Banton (1956), p.355 [80]	
	1931	Banton (1956), p.355 [80]	
	1921	Banton (1956), p.355 [80]	
	1911	Banton (1956), p.355 [80]	
**Monrovia** (Liberia)	1958	Fraenkel (1964), p.36 [48]	
	1939	Fraenkel (1964), p.33 [81]; Strong (1930), p.37 [26]	b)
**Abidjan** (Côte d'Ivoire)	1975	Antoine et al. (1987), p.105 [82]; Marguerat (1982), p.334 [83]	
	1955	Antoine et al. (1987), p.105 [82]; Bernus (1962), p.60 [84]	
	1936	Antoine et al. (1987), p.100 [82]; Le Pape (1993), p.338 [85]; Bouscayrol (1949) [86]; Soumahoro (1996) [87]	b)
**Bouaké** (Côte d'Ivoire)	1969	Marguerat (1982), p.333 [83]	
	1958	Marguerat (1982), p.333 [83]	
	1931	Marguerat (1982), p.333 [83]; Chaveau (1987), p.135 [88]	c)

Table TS2-2. The ethnically partitioned censuses we used in our study, by the order they appear in Dataset S1, and supporting references.

Notes: a) For these years, no ethnically partitioned censuses were available; we assumed the same ethnic group proportions of the nearest census to the future; **b)** For these years, we obtained numbers for some ethnic groups from different sources, and we estimated the numbers for the remaining ethnic groups based on either: i) their proportions in the nearest census to the future; ii) statements about their numbers in nearby years from various sources; **c)** In this case we estimated the numbers based on both the nearest census to the future, and statements on immigration patterns of the city. In several of these cases, we built several scenarios for the ethnic group numbers for which we lacked precise information (see Dataset S1).

5. Additional demographic information

In addition to obtaining ethnically partitioned censuses of cities, we obtained data on their population trends (Figure 2 of the main article), sex ratios, proportion of adults, and other relevant demographic variables, which were important for our simulations. The sources consulted to obtain demographic information are listed in Table TS2-3. They partly overlap with the sources listed in Table TS2-2.

City	Refs. of sources on demography
Kinshasa	63–64,89–96
Brazzaville	66,67,96–98
Bangui	33,68,96,99–101
Libreville	69,96
Douala	70–72,96
Yaoundé	73,96,102,103
Bissau	74–76,96
Conakry	77,78,96
Freetown	79,80,96
Monrovia	26,81,96
Abidjan	82–87,96,104–106
Bouaké	83,88,96,105,106

Table TS2-3. The cities under study, and the references of sources consulted for demographic information.

6. Hunting of apes and monkeys

We gathered information about practices of ape/monkey hunting from the same ethnographic sources used for the circumcision study, and we constructed Supplementary Table S3. We recorded all statements (mostly from before 1960) that explicitly referred to hunting of/feeding on: i) chimpanzees and/or gorillas (for Central Africa); ii) any monkeys (for West Africa). For some ethnic groups/populations, killing of the relevant animals was practiced without consuming their meat (e.g., the Aka Pygmies killed apes only to supply bushmeat to others [107–110]; the Tutsi and Hutu did it only to protect crops [16,111]; the Ngando killed bonobos rarely, only to obtain skins and bones [112]). Ape bushmeat consumption was inexistent/very rare among: peoples living in grasslands or non-forested highlands (Shi-

Havu, Tutsi, Hutu, Bamileke) because of rich agriculture and/or abundant livestock [16,111–115]; coastal peoples with longstanding contacts with Europeans (e.g., the Mpongwe [116,117]); Aka, Baka, and Twa Pygmies [107–110,115]; peoples heavily relying on fishing (e.g., the Pomo, Bomali, and Kaka living near Ouesso [118]). However, the vast majority of peoples living in chimpanzee and gorilla ranges killed and consumed these animals (Supplementary Table S3).

The remaining sources listed in the reference list [119–196] are additional ethnographic and other sources referred in Supplementary Table S3 and/or in the tables of Dataset S1.

References

1. Murdock GP (1967) Ethnographic Atlas. Pittsburgh: Univ of Pittsburgh Press.

2. Gray JP, editor (1999) Ethnographic Atlas Revised by World Cultures. New York: York College, CUNY.

3. Gordon RGJ, editor (2005) Ethnologue: Languages of the World, Fifteenth edition. Dallas: SIL International. Available: http://www.ethnologue.com. Accessed 2009 Sep 10.

4. Macro International (2009). Demographic and Health Surveys. Calverton: Macro International. Available: http://www.measuredhs.com. Accessed 2010 Feb 25.

5. Hewlett BS, Barnett D, Hooks V. Unpublished compilation of circumcision data, based on an unpublished ethnographic database constructed by George Peter Murdock before 1959, provided by and cited with permission of Professor Barry S Hewlett (Washington State Univ, Vancouver).

6. Baumann H, Westermann D (1962) Les peuples et les civilizations de l'Afrique. Paris: Payot.

7. Frobenius L, Von Wilm R (1929) Atlas Africanus: Belege zur Morphologie der afrikanischen Kulturen. München: C H Beck.

8. Tessmann G (1913) Die Pangwe: Völkerkundliche Monographie eines westafrikanischen Negerstammes. Berlin: Ernst Wasmuth.

9. Bruel G (1935) La France Équatoriale Africaine: le pays, les habitants, la colonization, les pouvoirs publics. Paris: Larose Éditeur.

10. Gaud F, Van Overbergh C (1911) Les Mandjia (Congo Français) [Vol VIII in the series Collection de Monographies Ethnographiques]. Brussels: Albert de Wit and Internationaal Instituut voor Bibliografie.

11. Friedrichs A (Herzogs zu Mecklenbourg), editor (1924) Wissenschaftliche Ergebnisse der Deutschen Zentral-Afrika Expedition 1907–1908. Leipzig: Klinkhardt & Biermann.

12. Halkim J (1911) Les Ababua [Vol VII in the series Collection de Monographies Ethnographiques]. Brussels: Albert de Wit and Internationaal Instituut voor Bibliografie.

13. Callone-Beaufaict A (1921) Azande: introduction à une ethnographie générale des bassins de l'Ubangi-Uele et de l'Aruwimi. Brussels: Maurice-Lamertin.

14. Burssens H (1958) Les peuplades de l'entre Congo-Ubangi (Ngbandi, Ngbaka, Mbanja, Ngombe et Gens d'Eau) [Vol 4 in the series Monographies Ethnographiques, Sciences de l'Homme]. Tervuren, Belgium: Annalen van het Koninklijk Museum van Belgisch-Congo.

15. Van Overbergh C, De Jonghe E (1907) Les Bangala (État Indépendant du Congo) [Vol I in the series Collection de Monographies Ethnographiques]. Brussels: Albert de Wit and Internationaal Instituut voor Bibliografie.

16. Frazer JG (1938) Native races of Africa and Madagascar. London: Percy Lund Humphries & Co.

17. Ferreira Diniz JO (1918) Populações Indígenas de Angola. Coimbra, Portugal: Imprensa da Universidade.

18. Bentley WH (1887) Life on the Congo. London: The Religious Tract Society.

19. Cureau AL (1915) Savage Man in Central Africa. London: Adelphi Terrace.

20. Berlin I (1996) From Creole to African: Atlantic Creoles and the Origins of African- American Society in Mainland North America. The William and Mary Quarterly (Third Series) 53: 251–288.

21. Köbben A (1956) Études Éburnéennes; Le Planteur Noir. Abidjan: Inst Français d'Afrique Noire, Centre de Côte d'Ivoire.

22. Paulme D (1962) Une societé de Côte d'Ivoire hier et aujourd'hui: les Bété. Paris: Mouton & Co.

23. Zaborowski M (1896) La circoncision: ses origines et sa répartition en Afrique et a Madagascar. L'Anthropologie 7: 653–675.

24. Labouret H (1914) Notes contributives a l'étude du peuple Baoulé. Revue Ethnogr Sociol 5: 83–91; 181–94.

25. Delafosse M (1893) Les Agni (Pai-Pi-Bri). L'Anthropologie 4: 402-45.

26. Strong RP, editor (1930) The African Republic of Liberia and the Belgian Congo, Based on the Observations Made and Material Collected During the Harvard African Expedition, 1926–27. Cambridge, MA: Harvard Univ Press.

27. Schwab C (1947) Tribes of the Liberian hinterland: Report of the Peabody Museum expedition to Liberia. Cambridge, MA: Peabody Museum of American Archaeology and Ethnology.

28. Biyi E (1929–30) The Kru and related peoples, West Africa. J Roy Afr Soc 29: 71–77 (1929); 29: 181–188 (1930).

29. Carreira A (1961) Organização social e económica dos povos da Guiné Portuguesa. Boletim Cultural da Guiné Portuguesa 16: 641–736. Referred in Dataset S1 as Carreira1961b.

30. Carvalho Viegas LA (1936–40) Guiné Portuguesa [Vol I: 1936; Vol II: 1939; Vol III: 1939–40]. Lisboa: Ministério do Ultramar e Colónia Portuguesa da Guiné.

31. Niang CI, Boiro H (2007) ''You Can Also Cut My Finger!'': Social Construction of Male Circumcision in West Africa, A Case Study of Senegal and Guinea-Bissau. Reprod Health Matters 15: 22–32.

32. Machado AJM (1972) Gentes de Catió. Geographica 8: 3–32.

33. Lebeuf JP (1954) Bangui (Oubangui-Chari, A.E.F.). Paris: Éditions de l'Union Française.

34. Moiya KN (1983) La circoncision chez les Ngbaka. Revista do Centro de Estudos Africanos da Universidade de São Paulo 6: 3–33.

35. Pépin J, Plamondon M, Alves AC, Beaudet M, Labbé AC (2006) Parenteral transmission during excision and treatment of tuberculosis

and trypanosomiasis may be responsible for the HIV-2 epidemic in Guinea-Bissau. AIDS 20: 1303–1311.

36. Bangna CN (2007) Características Sócio-Comportamentais Relacionadas ao Risco de Transmissão do HIV em Escolares de 15 a 24 anos, na Guiné-Bissau. Rio de Janeiro: Fundação Oswaldo Cruz, Escola Nacional de Saúde Pública Sérgio Arouca.

37. Drain PK, Halperin DT, Hughes JP, Klausner JD, Bailey RC (2006) Male circumcision, religion, and infectious diseases: an ecologic analysis of 118 developing countries. BMC Infect Dis 6: 172. Available: http://www.biomedcentral.com/1471-2334/6/172.

38. Sobral Gonçalves MJ (1973) Forros de São Tomé. Geographica 9: 51–78.

39. Kaplan S (1986) The Africanization of Missionary Christianity: History and Typology. J of Religion in Africa 16: 166–186.

40. Laburthe-Tolra P (1985) Initiations et societés secrètes au Cameroun: Essai sur la religion beti. Paris: Éditions Karthala.

41. Laburthe-Tolra P (1999) Vers la Lumière? Ou le Désir d'Ariel: a propos des Beti du Cameroun, Sociologie de la Conversion. Paris: Éditions Karthala.

42. Bureau R (1996) Le peuple du fleuve: sociologie de la conversion chez les Duala. Paris: Éditions Karthala.

43. Van Wing J (1938) Études Bakongo: Sociologie, Religion et Magie. Brussels: Van Campenhout.

44. Raponda-Walker A, Sillans R (1962) Rites et croyances des peuples du Gabon. Paris: Présence Africaine.

45. Ittmann J (1953) Volkskundliche und religiöse Begriffe im nördlichen Waldland von Kamerun. Berlin: Dietrich Reimer.

46. Rebuffat E (1927) Contribution à l'étude du relèvement de la natalité au Congo Belge [dissertation]. Instituut voor Tropische Geneeskunde, Antwerp, ITG/Examen B-48a.

47. Peltzer K, Nqeketo A, Petros G, Kanta X (2008) Traditional circumcision during manhood initiation rituals in the Eastern Cape, South Africa: a pre-post intervention evaluation. BMC Public Health 8: 64. Available: http://www.biomedcentral.com/1471-2458/8/64.

48. Decorse GJ (1906) Du Congo au lac Tchad: la brousse telle quelle est, et les gens tels qu'ils sont (Mission Chari-Lac Tchad 1902–1904). Paris: Augustin Challamel.

49. Forde D, editor (1954) Peoples of the Central Cameroons. London: International African Institute.

50. Hoover JJ (1978) Mythe et Remous Historique: A Lunda Response to De Heusch. History in Africa 5: 63–80.

51. Millous P (1936) Notes sur l'anthropologie des Baya du Cameroun, en 1935, et sur l'ethnologie des Baya de la Haute-Mambéré (A.E.F.), en 1905. L'Anthropologie 46: 91–99.

52. Dugast I (1954) The Banen, Bafia and Balom of the French Cameroons. In: Forde D, editor. Peoples of the Central Cameroons. London: International African Institute.

53. Van Geluwe H (1960) Les Bali et les peuplades apparantées (Ndaka-Mbo-Beke-Lika-Budu-Nyari) [Vol 5 in the series Monographies Ethnographiques, Sciences de l'Homme]. Tervuren, Belgium: Annalen van het Koninklijk Museum van Belgisch-Congo.

54. Maclaud C (1907) Notes anthropologiques sur les Diola de la Casamance. L'Anthropologie 18: 69–98.

55. Delafosse M (1912) Haut-Sénégal-Niger: le pays, les peuples, les langues. Paris: Larose.

56. Meireles AM (1960) Mutilações étnicas nos Manjacos. Bissau: Centro de Estudos da Guiné Portuguesa.

57. Hulstaert G (1938) Le Marriage des Nkundo. Brussels: Van Campenhout.

58. Koch CWH (1913) Die Stämme des Bezirks Molundu: in Sprachlicher, Geschichtlicher und Völkerkundlicher Beziehung. Bässler-Archiv: Beiträge zu Völkerkunde 3: 257–312.

59. Delafosse M, Poutrin L (1930) Enquête coloniale dans l'Afrique Française Occidentale et Équatoriale, sur l'organization de la famille indigène, les fiançailles, le mariage, avec une esquisse générale des langues de l'Afrique. Paris: Soc d'Éditions Géographiques, Maritimes et Coloniales.

60. Saint Moulin L (2003) Conscience nationale et identités ethniques: contribution à une culture de la paix. Congo-Afrique 372: 93–128.

61. Inforcongo (1959) Belgian Congo. Brussels: Office de l'Information et des Relations Publiques pour le Congo Belge et le Ruanda-Urundi.

62. Institut Cartographique Militaire (1921) Carte Administrative du Congo Belge. Brussels: Institut Cartographique Militaire. Afrika-Archief (Federale Overheidsdients – Buitenlandse Zaken, Buitenlandse Handel en Ontwikkelingssamenwerking (FO-BZBHO), Brussels), Inventory Cartes, Folder 16.

63. Spitaels G (1959) Letter to the Centre de Recherches et d'Informations Socio-Politiques (Brussels), dated from October 21, 1959 [contains a census of Leopoldville, discriminating regions of origin of inhabitants]. Archives of Afrika Museum (Tervuren, Belgium).

64. Affaires Indigènes et Main d'Oeuvre (AIMO) (1927) Enquête sur la Main d'œuvre, District Urbain de Leopoldville. Leopoldville, Belgian Congo: AIMO, Province du Congo-Kasai. Afrika Archief (FO-BZBHO, Brussels), Series GG, Folder GG 16186.

65. Douchet S, Dubois A (1911–12) Rapport sur le fonctionnement du lazaret pour trypanosés de Leopoldville pendant l'année [1911 through 1912]. Leopoldville, Belgian Congo. Afrika Archief (FO-BZBHO, Brussels), Inventory A39, Box 80.

66. Balandier G (1955) Sociologie des Brazzavilles Noires. Paris: Librairie Armand Colin.

67. Subdivision Autonome de Brazzaville (1932) Rapport Annuel, Subdivision Autonome de Brazzaville. Brazzaville, French Congo: Colonie du Moyen-Congo, Afrique Équatoriale Française. Centre des Archives d'Outre-Mer (CAOM), Generic AEF, Series 4D, Box 4(2) D 54.

68. Soret M (1961) Bangui: étude socio-démographique de l'habitat. Brazzaville: ORSTOM and Institut de Recherches.

69. Lasserre G (1958) Libreville: la ville et sa région (Gabon –AEF). Paris: Librairie Armand Colin.

70. Mainet G (1985) Douala: Croissance et Servitudes. Paris: Éditions L'Harmattan.

71. Service de Santé (1935) Rapport du Service de Santé, Division du Wouri, Territoire du Cameroun. Douala, Cameroon: Service de Santé. IMTSSA, Box 481.

72. Gouellain R (1975) Douala: ville et histoire. Paris: Institut d'Ethnologie.

73. Roubaud F (1994) La question ethnique sur le marché de travail à Yaoundé: discrimination ou solidarité? Paris: DIAL, ORSTOM.

74. Carreira A (1962) Guiné Portuguesa: população autóctone segundo os recenseamentos para fins fiscais. Bissau: Imprensa Portuguesa.

75. Junta de Investigação do Ultramar (1950) Província da Guiné – Censo da População. Lisboa: Centro de Estudos Políticos e Sociais.

76. Província da Guiné (1940) Anuário da Guiné Portuguesa. Bissau, Portuguese Guinea: Província da Guiné.

77. Goerg O (1990) La genèse du peuplement de Conakry. Cahiers d'Études Africaines 31: 73–99.

78. Goerg O (2006) Chieftainships between Past and Present: From City to Suburb and Back in Colonial Conakry, 1890s-1950s. Africa Today 52: 3–27.

79. Harvey ME (1971) Social Change and Ethnic Relocation in Developing Africa: the Sierra Leone Example. Geografiska Annaler Series B 53:94–106.

80. Banton M (1956) Adaptation and integration in the social system of Temne immigrants in Freetown. J Int Afr Inst 26: 354–368.

81. Fraenkel M (1964) Tribe and Class in Monrovia. London: Oxford Univ Press.

82. Antoine P, Dubresson A, Manou-Savina A (1987) Abidjan «côté cours». Paris: Éditions Karthala and Éditions de l'ORSTOM.

83. Marguerat Y (1982) Des ethnies et des villes: Analyse des migrations vers les villes de Côte d'Ivoire. Cahiers de l'ORSTOM, Série Sci Hum 18: 303–340.

84. Bernus E (1962) Abidjan: Note sur l'agglomération d'Abidjan et sa population. Bull de l' Inst Français d'Afrique Noire (IFAN) 24: 54–85.

85. Le Pape M (1993) L'attraction urbaine: soixante-cinq ans d'observations sur Abidjan. Cahiers Sci Hum 29: 333–348.

86. Bouscayrol R (1949) Notes sur le peuple Ebrié. Bull de l'IFAN 11: 382–408.

87. Soumahoro C (1996) Formes et rythmes d'expansion de l'habitat locatif populaire à Abidjan (1920–1992) [doctoral thesis]. Univ Pierre Mendès France, Grenoble II, France.

88. Chaveau JP (1987) La part Baulé: effectif de population et de domination ethnique: une perspective historique. Cahiers d'Études Africaines 27: 123–165.

89. Duren A (1950) Quelques données sue la situation démographique de la cité indigène de Leopoldville entre 1923 et 1947. Bull Séances Inst Roy Colon Belg 21: 708–717.

90. Pain M (1984) Kinshasa: la ville et la cité. Paris: Éditions de l'ORSTOM.

91. Affaires Indigènes et Main d'Oeuvre (AIMO) (1925–52) Rapport Annuel, District Urbain the Leopoldville [1925 through 1952]. Leopoldville, Belgian Congo: AIMO, Province du Congo-Kasai. Afrika Archief (FO-BZBHO, Brussels), Inventory A39, Boxes RA/AIMO 119 and RA/AIMO 120.

92. Service de l'Hygiène Publique (1933–58) Rapport sur l'hygiène publique au Congo Belge pendant l'année [1933 through 1958]. Leopoldville, Belgian Congo: Service de l'Hygiène Publique.

93. Service de l'Hygiène Publique (1930–39) Rapport Annuel, District Urbain de Leopoldville [1930 through 1939]. Leopoldville, Belgian Congo: Service de l'Hygiène Publique. Afrika Archief (FO-BZBHO, Brussels), Inventory A39, Box RA/MED 46.

94. Mouchet R (1913) La tuberculose à Leopoldville (Congo Belge). Bull Soc Pathol Exot Fil 6: 55–68.

95. Service Médical (1919) Letter from the head of the Service Médical of the Congo-Kasai province to the governor, July 26, 1919. Leopoldville, Belgian Congo. Afrika Archief (FO-BZBHO, Brussels), Series GG, Folder GG 16864.

96. Lahmeyer J (2006) Population Statistics: historical demography of all countries, their divisions and towns. Available: http://www.populstat.info. Accessed 2009 Aug 22.

97. Auger A (1972) Le ravitaillement vivrier traditionnel de la population africaine de Brazzaville. In: Centre National de la Recherche Scientifique (CNRS), editor. La croissance urbaine en

Afrique noire et a Madagascar [in the series Sciences humaines, Colloques internationaux du CNRS]. Paris: Éditions du CNRS.

98. Service de Santé (1930–34) Rapport annuel du Chef du Service de Santé, Colonie du Moyen-Congo, Afrique Équatoriale Française [1930 through 1934]. Brazzaville, AEF: Service de Santé. IMTSSA, Box 117.

99. Boulvert Y (1986) Aperçu bibliographique sur l’évolution de la population du Centrafrique et de la capitale Bangui. Bangui: Centre ORSTOM de Bangui.

100. Boulvert Y (1989) Bangui (1889–1989): Points de Vue et Témoignages. Paris: Ministère de la Coopération et du Développement.

101. Service de la Statistique (1964) Annuaire Statistique 1957–1962, République Centrafricaine. Bangui, Central African Republic: Service de la Statistique.

102. Franqueville A (1968) Le paysage urbain de Yaoundé. Les Cahiers d’Outre-Mer 82: 113–154.

103. Franqueville A (1984) Yaoundé: construire une capitale. Paris: Éditions de l’ORSTOM.

104. Le Pape M (1985) De l’espace et des races à Abidjan, entre 1903 et 1934. Cahiers d’Études Africaines 25: 295–307.

105. Domergue-Cloarec D (1986) La Santé en Côte d’Ivoire, 1905–1958. Toulouse: Association des Publications Univ Toulouse-Le Mirail.

106. Cotten AM, Marguerat Y (1978) Deux réseaux urbains Africains, Cameroun et Côte d’Ivoire: la mise en place des réseaux urbains. Cahiers d’Outre-Mer 116: 348–385.

107. Despois J (1946) Les genres de vie des populations de la forêt dans le Cameroun oriental. Ann Géogr 55: 19–38.

108. Lewis J (2002) Forest Hunther-gatherers and Their World: A Study of the Mbendjele Yaka Pygmies of Congo-Brazzaville and Their Secular and Religious Activities and Representations [doctoral thesis]. Department of Social Anthropology, London School of Economics and Political Science, London.

109. Hattori S (2005) Nature conservation and hunter-gatherer’s life in Cameroonian rainforest. Afr Study Monogr 29: Suppl 41–51.

110. Köhler A (2005) Of Apes and Men: Baka and Bantu Attitudes to Wildlife and the Making of Eco-Goodies and Baddies. Conservation and Society 3: 407–435.

111. Sicotte P, Uwengeli P (2002) Reflections on the concept of nature and gorillas in Rwanda: Implications for conservation. In: Fuentes A, Wolfe L, editors. Primates Face to Face: The Conservation Implications of Human-Nonhuman primate Interconnections. Cambridge: Cambridge Univ Press.

112. Lingomo B, Kimura D (2009) Taboo of eating Bonobo among the Bongando people in the Wamba region, Democratic Republic of Congo. Afr Study Monogr 30: 209–225.

113. Jones JE (2006) “Gorilla Trails in Paradise”: Carl Akeley, Mary Bradley, and the American Search for the Missing Link. J Amer Culture 29: 321–336.

114. Bradley MH (1936) On the Gorilla Trail. New York: D. Appleton Century.

115. Fossey D (1983) Gorillas in the mist. New York: Penguin Books.

116. Burton RF (1865) A Day among the Fans. Trans Ethnol Soc Lond 3: 36–47.

117. Du Chaillu PB (1861) Explorations and Adventures in Equatorial Africa. New York: Harper & Brothers.

118. Bruel G (1910) Les populations de la Moyenne Sanga: les Pomo et les Boumali. Revue Ethnogr Sociol 1: 3–32.

119. Wolfe AW (1961) In the Ngombe Tradition: continuity and change in the Congo. Evanston: Northwestern Univ Press.

120. Meder A (1999) Gorillas in African Culture and Medicine. Gorilla J 18: 11–15.

121. Althabé G (1962) Problèmes socio-économiques du Nord-Congo. Cahiers de l’Institut de Science Économique Apliquée [series V] 5: 189–282.

122. Darré E (1923) La Tribu Bondjo, ses Mœurs, ses Coutumes. Bull Soc Rech Congolaises 3: 53–73.

123. Blake S (1993) A reconnaissance survey in the Likouala swamps of northern Congo and its implications for conservation [dissertation]. Univ of Edinburgh, Edinburgh.

124. Kano T, Asato R (1994) Hunting pressure on chimpanzees and gorillas in the Motaba river area, northeastern Congo. Afr Study Monogr 15: 143–162.

125. Ballif N (1992) Les Pygmées de la Grande Forêt. Paris: L'Harmattan.

126. Giles-Vernick T (2002) Cutting the Vines of the Past: Environmental Histories of the Central African Rain Forest. Charlottesville: Univ Press of Virginia.

127. Bennett AL (1899) Ethnographical notes on the Fang. J Roy Anthr Inst Great Britain Ireland 29: 66–98.

128. Trezenem E (1936) Notes ethnographiques sur les tribus Fan du Moyen-Ogooué. J Soc Africanistes 6: 65–93.

129. Merfield FG (1957) Gorillas were my Neighbours. London: The Company Book Club.

130. Alexandre P, Binet J (1958) Le groupe dit Pahouin (Fang-Boulou-Beti). Paris: L'Harmattan.

131. Walker A (1924) Les tribus du Gabon. Bull Soc Rech Congolaises 4: 55–99.

132. Allys P (1930) Monographie de la tribu des Dzems (Ngoko-Sangha). Bull Soc Rech Congolaises 11: 3–21.

133. Dugast I (1955–60) Monographie de la tribu des Ndiki (Banen du Cameroun) [Vol I (1955): Untitled; Vol II (1960): Vie sociale et familiale]. Paris: Institut d'Ethnologie.

134. Delacour A (1912–13) Les Tenda (Koniagui, Bassari, Badyaranké) de la Guinée Française. Revue Ethnogr Sociol 3: 287–296; 370–381 (1912); 4: 31–52; 105–120; 140–153 (1913).

135. Mendes FA (1948) Vida material dos Brâmes. Boletim Cultural da Guiné Portuguesa 3: 81–113.

136. Machat J (1906) Les Rivières du Sud et le Fouta-Diallon: Géographie physique et Civilizations indigènes. Paris: Augustin Challamel.

137. Shattuck GC (1929) Liberia and the Belgian Congo. Geogr J 73: 216–238.

138. Hallouin C (1947) Géographie humaine de la subdivision de Daloa. Bull de l'IFAN 9: 18–55.

139. Augé M (1975) Théorie des pouvoirs et idéologie: étude de cas en Côte d'Ivoire. Paris: Hermann.

140. Tate GHH (1942) The Lower Cavally River, West Africa. Geogr Rev 32: 574–584.

141. Jensen AE (1933) Beschneidung und Reifezeremonien bei Naturvölkern. Stuttgart: Strecker und Schröder.

142. Laurent C, Bourgeois A, Mpoudi M, Butel C, Peeters M, et al. (2004) Commercial Logging and HIV Epidemic, Rural Equatorial Africa. Emerg Infect Dis 10: 1953–1956.

143. Lagarde E, Schim van der Loeff M, Enel C, Holmgren B, Dray-Spira R, et al. (2003) Mobility and the spread of human immunodeficiency virus into rural areas of West Africa. Int J Epidemiol 32: 744–752.

144. Auvert B, Buvé A, Lagarde E, Kahindo M, Chege J, et al. (2001) Male circumcision and HIV infection in four cities in sub-Saharan Africa. AIDS 15: Suppl 4S31–40.

145. Awusabo-Asare K, Biddlecom A, Kumi-Kyereme A, Patterson K (2006) Adolescent Sexual and Reproductive Health in Ghana: Results from the 2004 National Survey of Adolescents [Occasional report no.22]. New York: Guttmacher Institute.

146. Torday E (1928) Dualism in Western Bantu Religion and Social Organization. J Roy Anthr Inst Great Britain Ireland 58: 225–245.

147. Torday E, Joyce TA (1906) Notes on the Ethnography of the Ba-Yaka. J Roy Anthr Inst Great Britain Ireland 36: 39–59.

148. Torday E, Joyce TA (1907) Note on the Southern Ba-Mbala. Man 7: 81–84.

149. Weeks JH (1914) Among the Primitive Bakongo. London: Seeley, Service & Co.

150. Guiral L (1886) Les Batéké. Revue Ethnogr 5: 135–166.

151. Courboin A (1904–08) Les Populations de l'Alima: Congo Français. Bull Soc Roy Géogr Anvers 28: 273–308 (1904); 32: 648–70 (1908).

152. Borgonjon JF (1945) De besnijdenis bij de Tshokwe. Aequatoria 8: 13–25; 59–74.

153. Turner VW (1969) Symbolization and Patterning in the Circumcision Rites of Two Bantu-speaking Societies. In: Douglas M, Kaberry PM, Forde D, editors. Man in Africa. London: Tavistock Publications.

154. Janssens A (1912) La mission de Mpangu “St Pierre Claver”. ARCCIM Archives (Rome), Z/III/b/3/1/21.

155. Schmitz R (1912) Les Baholoholo [Vol IX in the series Collection de Monographies Ethnographiques]. Brussels: Albert de Wit and Internationaal Instituut voor Bibliografie.

156. Weeks JH (1909–10) Anthropological Notes on the Bangala of the Upper Congo River. J Roy Anthr Inst Great Britain Ireland 39: 97–136; 416–459 (1909); 40: 360–427 (1910).

157. Gilliard L (1928) Grammaire synthétique du Lontomba suivi d’un vocabulaire. Brussels: Éditions de l’Essorial.

158. Tanghe B (1948) La famille chez les Ngbandi. Révue de l’AUCAM 23: 80-82.

159. Landor AHS (1907) Across widest Africa: an account of the country and people of Eastern, Central and Western Africa as seen during a twelve months’ journey from Djibuti to Cape Verde. London: Hurst & Blackett.

160. De Mahieu W (1986) Qui a obstrué la cascade: analyse sémantique du rituel de la circoncision chez les Komo du Zaire. Paris: Éditions de la Maison des Sciences de l’Homme; Cambridge: Cambridge Univ Press.

161. Dedave A (1957) Les Pêcheurs de Stanleyville: Quelques Aspects de la Vie Sociale et Coutumière des Wagenia. J Int Afr Inst 27: 262–267.

162. Martins Vaz J (1970) No Mundo dos Cabindas: Estudo Etnográfico. Lisboa: Editorial LIAM.

163. Clarke R (1863) Sketches of the Colony of Sierra Leone and Its Inhabitants. Trans Ethnol Soc London 2: 320–363.

164. Migeod FWH (1925) A view of the colony of Sierra Leone. J Afr Soc 25: 1–9.

165. Bongaarts J, Reining P, Way P, Conant F (1989) The relationship between male circumcision and HIV infection in African populations. AIDS 3: 373–377.

166. Loyre E (1909) Les Populations de la Moyenne-Sangha. Questions Diplomatiques et Coloniales 28: 406–420.

167. Perrois L (1967) Peuples et civilizations de la grande forêt. La Cité 28: 86–95.

168. Clozel FJ (1896) Les Bayas: notes ethnographiques et linguistiques. Paris: Librairie Africaine et Coloniale.

169. Fontaine H (1928) La circoncision au village Baya de Kouki (Circonscription de l'Ouham). Bull Soc Rech Congolaises 9: 101.

170. Bryk F (1934) Circumcision in Man and Woman: its History, Psychology, and Ethnology. Honolulu: Univ Press of the Pacific.

171. Kerandel J (1909) Note sur la mission du Haut Logone. Ann Hyg Méd Colon 12: 107–114.

172. Laman K (1957) The Kongo (2nd volume). Uppsala: Almquist & Wiksells.

173. Zenker G (1895) Yaunde. Mittheilungen von Furschungsreisenden und Gelehrten aus den deutschen Schutzgebieten 8: 35–70.

174. Henric D (1902) Notes d'ethnographie et d'anthropologie sur les Haoussas (Afrique Centrale). Ann Hyg Méd Colon 5: 414–431.

175. Avelot R (1913) Notice historique sur les Ba-Kalé. L'Anthropologie 24: 197–240.

176. Nassau RH (1904) Fetichism in West Africa. New York: Charles Schribner's Sons.

177. Bureau R (1962) Ethno-sociologie des Douala et apparentés. Yaoundé: IRCAM.

178. Bekombo-Priso M (2009) Penser l'Afrique: regards d'un ethnologue Dwálá. Nanterre, France: Societé d'Ethnologie.

179. Malcolm LWG (1925) Notes on the Physical Anthropology of certain West African Tribes; Part 2: The Tribes of the Grassland Area, Central Cameroon. Wien: Selbstverlage der Anthropologischen Gesellschaft.

180. Nicol Y (1929) La Tribu des Bakoko: Étude Monographique dÉconomie Coloniale. Paris: Librairie Coloniale et Orientaliste Larose.

181. Egerton FC (1938) African Majesty: A Record of Refuge at the Court of the King of Bangangté in the French Cameroons. London: George Routledge & Sons.

182. Littlewood M (1954) The Bamileke of the French Cameroons. In: Forde D, editor. Peoples of the Central Cameroons. London: International African Institute.

183. Littlewood M (1954) The Bamum of the French Cameroons. In: Forde D, editor. Peoples of the Central Cameroons. London: International African Institute. Referred in Dataset S1 as Littlewood1954b.

184. Lecoq R (1953) Les Bamileke: une civilization africaine. Paris: Présence Africaine Éditions.

185. McCulloch M (1954) The Tikar of the British and French Cameroons. In: Forde D, editor. Peoples of the Central Cameroons. London: International African Institute.

186. Gausset Q (1999) Islam or Christianity? The Choices of the Wawa and the Kwanja of Cameroon. J Int Afr Inst 69: 257–278.

187. Porter AT (1953) Religious affiliation in Freetown, Sierra Leone. Africa 23: 3–14.

188. Carreira A (1961) Símbolos, ritualistas, e ritualismos animo-fetichistas na Guiné Portuguesa. Boletim Cultural da Guiné Portuguesa 16: 505–540.

189. Quintino FC (1969) Os povos da Guiné. Boletim Cultural da Guiné Portuguesa 24: 861–916.

190. Paulme D (1947) L' initiation des filles en pays Kissi (Haute-Guinée). In: Junta de Investigações Coloniais (JIC), editor. Segunda Conferência Internacional dos Africanistas Ocidentais. Lisboa: Ministério das Colónias, JIC.

191. Tully JJ (1994) The place of Islamic law in Sierra Leone. The Muslim World 84: 300–316.

192. Jedrej MC (1986) Dan and Mende masks: a structural comparison. Africa 56: 71–80.

193. Paulme D, editor (1971) Classes et associations d’age en Afrique de l’Ouest. Paris: Librairie Plon.

194. Niangoran-Bouah G (1964) La division du temps et le calandrier rituel des peuples lagunaires de Côte d’Ivoire. Paris: Institut d’Ethnologie.

195. Ruelle E (1904) Notes anthropologiques, ethnographiques et sociologiques sur quelques populations noires du 2éme Territoire Militaire de l’Afrique Occidentale Française. L’Anthropologie 15: 657–703.
